# Atomoxetine Effects on Executive Function as Measured by the BRIEF-A in Young Adults with ADHD: A Randomized, Double-Blind, Placebo-Controlled Study

**DOI:** 10.1371/journal.pone.0104175

**Published:** 2014-08-22

**Authors:** Lenard A. Adler, David B. Clemow, David W. Williams, Todd M. Durell

**Affiliations:** 1 Departments of Psychiatry and Child and Adolescent Psychiatry, New York University, New York, New York, United States of America; 2 Lilly USA, LLC, Indianapolis, Indiana, United States of America; 3 inVentiv Health Clinical, Indianapolis, Indiana, United States of America; Monash University, Australia

## Abstract

**Objective:**

To evaluate the effect of atomoxetine treatment on executive functions in young adults with attention-deficit/hyperactivity disorder (ADHD).

**Methods:**

In this Phase 4, multi-center, double-blind, placebo-controlled trial, young adults (18–30 years) with ADHD were randomized to receive atomoxetine (20–50 mg BID, N = 220) or placebo (N = 225) for 12 weeks. The Behavior Rating Inventory of Executive Function-Adult (BRIEF-A) consists of 75 self-report items within 9 nonoverlapping clinical scales measuring various aspects of executive functioning. Mean changes from baseline to 12-week endpoint on the BRIEF-A were analyzed using an ANCOVA model (terms: baseline score, treatment, and investigator).

**Results:**

At baseline, there were no significant treatment group differences in the percentage of patients with BRIEF-A composite or index T-scores ≥60 (*p>*.5), with over 92% of patients having composite scores ≥60 (≥60 deemed clinically meaningful for these analyses). At endpoint, statistically significantly greater mean reductions were seen in the atomoxetine versus placebo group for the BRIEF-A Global Executive Composite (GEC), Behavioral Regulation Index (BRI), and Metacognitive Index (MI) scores, as well as the Inhibit, Self-Monitor, Working Memory, Plan/Organize and Task Monitor subscale scores (*p*<.05), with decreases in scores signifying improvements in executive functioning. Changes in the BRIEF-A Initiate (*p* = .051), Organization of Materials (*p* = .051), Shift (*p* = .090), and Emotional Control (*p* = .219) subscale scores were not statistically significant. In addition, the validity scales: Inconsistency (*p* = .644), Infrequency (*p* = .097), and Negativity (*p* = .456) were not statistically significant, showing scale validity.

**Conclusion:**

Statistically significantly greater improvement in executive function was observed in young adults with ADHD in the atomoxetine versus placebo group as measured by changes in the BRIEF-A scales.

**Trial Registration:**

ClinicalTrials.gov NCT00510276

## Introduction

A growing body of evidence suggests that attention-deficit/hyperactivity disorder (ADHD) consists of more than just its primary diagnostic symptoms of inattention, impulsiveness, and hyperactivity. Some researchers argue that symptoms of deficient executive functioning (EF) are also involved in ADHD symptomology and consider ADHD a disorder of executive functioning dysregulation [1], [2]. There are 2 ADHD dimensions believed to be important to executive functioning: the inattentive dimension (working memory, planning/problem-solving) and the hyperactive, impulsive dimension (behavioral inhibition, motor regulation) [3], [4]. One group of researchers has grouped executive functioning into 6 clusters, all of which relate to core ADHD symptoms: activation (organizing tasks and materials, prioritizing and starting tasks, and estimating time); focus (sustaining focus, shifting focus to different tasks); effort (regulating alertness, sustaining effort, and processing speed); emotion (managing/controlling frustration and emotion); memory (utilizing working memory and accessing recall); and action (recognizing appropriate behavior in response to a situation, self-regulating action, and impulsivity) [4], [5].

Executive functions become increasingly important as children mature and are expected to assume more responsibility for managing their behavior. Multiple studies have shown that children with ADHD are more likely to have executive function impairments that persist into adulthood [4], with neuropsychological studies consistently finding adults with ADHD to have impairments on measures of executive function [6]. Deficits in any one aspect of executive functioning might contribute to impairment, some of which could be associated with ADHD [3].

Response inhibition, working memory, and other executive functioning activities involve brain circuits that prioritize, integrate, and regulate other cognitive functions such as attention, motivation, and emotion. Many of these functions are controlled via regions of the brain that are known to have neurobiological differences in patients with ADHD compared to those without ADHD, and areas of the brain involved in executive function have neuronal projections that connect these different brain regions [7], [8], [9], [10]. The executive function areas of the brain and the circuits connecting them to the brainstem and other functional areas are heavily innervated with dopaminergic, serotonergic, noradrenergic, and cholinergic neurons. Dysregulation in these neurotransmitter signaling systems is believed to be involved in the manifestation of ADHD [7], [11], [12], [13].

Atomoxetine hydrochloride is a nonstimulant, selective norepinephrine reuptake inhibitor approved in the US as a treatment for ADHD in children, adolescents, and adults, as defined by the Diagnostic and Statistical Manual of Mental Disorders, Fourth Edition-Text Revision (DSM-IV-TR) [14]. Atomoxetine was approved for use in adults (age 18 and older) with ADHD based on 2 pivotal, randomized, placebo-controlled trials [15]. In these studies, the primary outcome measure was the Total ADHD Symptom Score, which is the sum of the Inattention and Hyperactivity/Impulsivity subscales of the investigator-rated Conners’ Adult Attention Rating Scale (CAARS). For the CAARS, each of the 18 items of the subscales corresponds to one of the 18 DSM-IV-TR symptoms for ADHD.

In a phase 4 study that focused on prospectively assessing the efficacy and safety of atomoxetine in young adults, atomoxetine was observed to reduce ADHD symptoms and improve quality of life in young adults compared with placebo [16]. In the original publication that presents the primary efficacy measure results (CAARS), an *a priori* secondary analysis of Behavior Rating Inventory of Executive Function-Adult (BRIEF-A) data was also noted: after 12-weeks, atomoxetine-treated patients had statistically significantly greater mean reductions from baseline in BRIEF-A Global Executive Composite (GEC) score than placebo-treated patients [16]. However, additional BRIEF-A results were not provided. Therefore, the purpose of this paper is to fully examine the BRIEF-A data and present the BRIEF-A results from this ADHD study, including results from all of the subscales, examining the effect of atomoxetine on executive functioning in young adult ADHD patients.

## Materials and Methods

The protocol for this trial and supporting CONSORT checklist are available as supporting information; see Checklist S1 and Protocol S1. The study procedures have been described previously in detail [16] and thus are summarized within this paper. This study was a phase 4, multicenter, 12-week, randomized, placebo-controlled, double-blind trial conducted at 32 sites in the United States and Puerto Rico between August 2007 and February 2009. Participants completing the double-blind portion of the trial were eligible to enter an optional 12-week, open-label, extension period. This paper reports the results of an *a priori* secondary analysis of BRIEF-A data from the 12-week double-blind acute period.

### Ethics Statement

Before initiation of any study procedures, site investigators provided participants with thorough verbal and written descriptions of protocol requirements, and participants gave written consent under procedures approved by each participating site’s institutional review board, which included: New England Institutional Review Board (IRB), University of Kentucky IRB, Western IRB, New York University School of Medicine Institutional Board of Research Associates, University of Nebraska Medical Center IRB, University of Cincinnati Medical Center IRB, McLean Hospital IRB, and Copernicus Group IRB. The study was conducted in accordance with the ethical principles originating from the Declaration of Helsinki and consistent with Good Clinical Practices.

### Participant Population

**Figure 1:**
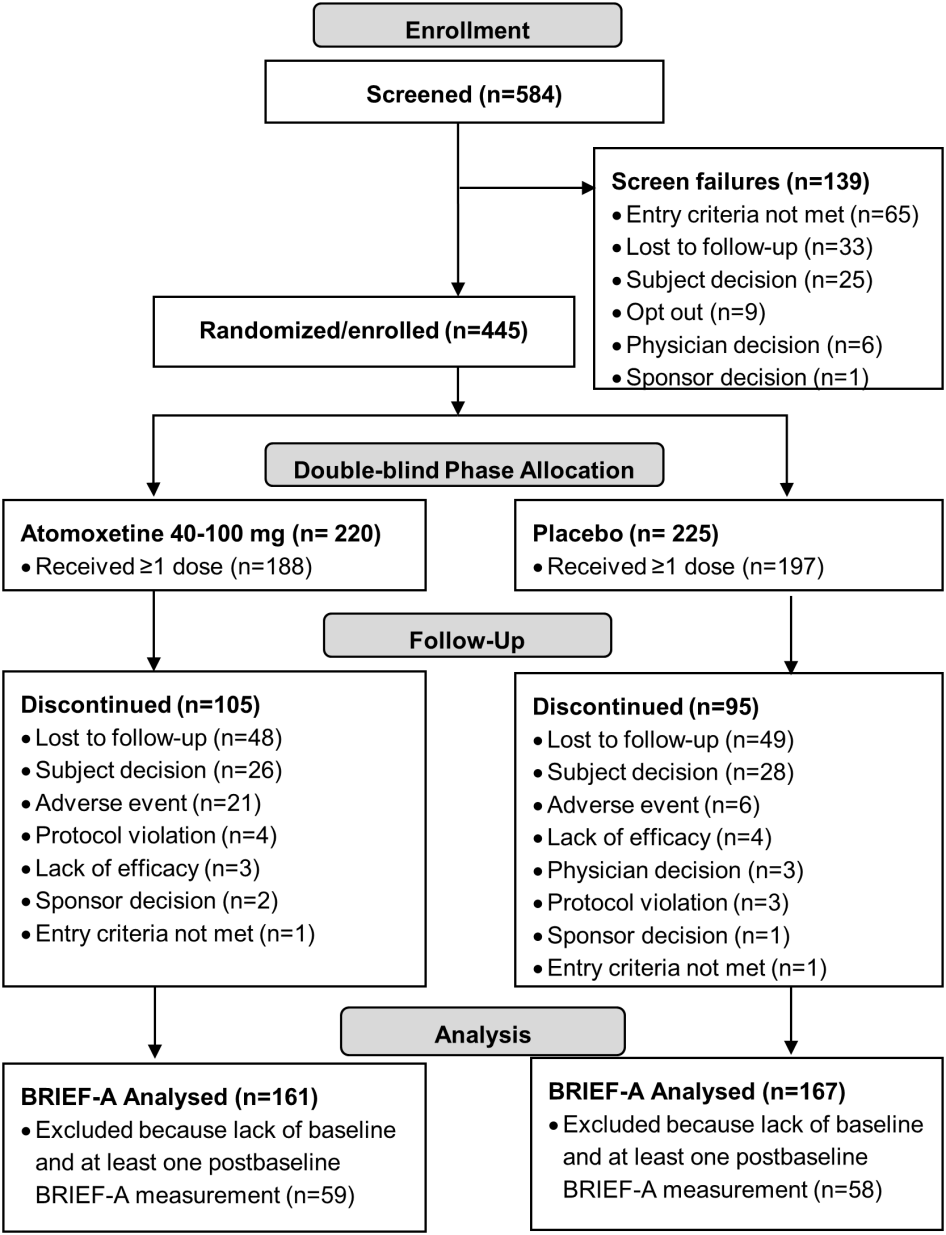
Participant disposition flow diagram.

Young adults (18–30 years of age) with ADHD were randomized 1∶1 to the atomoxetine or placebo group for 12 weeks. Figure 1 details patient flow through the study. Participants met DSM-IV-TR criteria for ADHD, as determined by a clinical interview and as assessed by the Adult ADHD Clinical Diagnostic Scale (ACDS) version 1.2. The ACDS v1.2 is a validated, structured diagnostic scale for adult ADHD, containing separate modules of assessment of childhood and adulthood ADHD symptoms and impairments, which establishes whether a DSM-IV-TR diagnosis of adult ADHD is present or not [17]. Details of patient enrollment, allocation, and follow-up have been described previously in detail [16]. Of the 584 participants screened, 445 were randomized to treatment (220 atomoxetine; 225 placebo), and 385 received at least 1 dose of study drug. For this paper, which is focused on BRIEF-A results, only patients with baseline and at least one postbaseline BRIEF-A measurement were included in analyses: atomoxetine (N = 161) and placebo (N = 167). For each analysis, table footnotes provide details of the number of patients included for each analysis.

Participants with concomitant current (if diagnosed greater than 6 months immediately prior to Visit 1) or lifetime diagnoses of specific phobias, generalized anxiety disorder, or social anxiety disorder were allowed in the trial, as were participants with a current (if diagnosed greater than 2 years immediately prior to Visit 1) or lifetime history of dysthymia. Potential participants were excluded from the trial if they had current major depression, panic disorder, post-traumatic stress disorder, an eating disorder, or substance abuse or dependence, as well as current or lifetime obsessive-compulsive disorder, bipolar disorder, or psychosis. Concomitant medications such as anxiolytics and antidepressants with primarily central nervous system activity were not allowed, with few exceptions (e.g., acute analgesics). The incidence of prior stimulant medication use was approximately 35–38% and similar between treatment groups (Table 1).

**Table 1:** Patient Baseline Characteristics.

	Atomoxetine	Placebo	*P*-value
Number of participants (N)^a^	220	225	
Age, years (mean ± SD)	24.7±3.4	24.7±3.5	.922^b^
Gender, n (%)			.774^c^
Female	92 (41.8)	98 (43.6)	
Male	128 (58.2)	127 (56.4)	
Height, cm (mean ± SD)^b^	171.9±9.6	172.5±9.9	.514^b^
Weight, kg (mean ± SD)^b^	79.6±22.6	81.6±21.5	.351^b^
Ethnicity, n (%)			.052^c^
African descent	12 (5.5)	26 (11.6)	
White	169 (76.8)	166 (73.8)	
East Asian	8 (3.6)	7 (3.1)	
Hispanic	27 (12.3)	25 (11.1)	
Native American	0 (0.0)	1 (0.4)	
West Asian	4 (1.8)	0 (0.0)	
DSM-IV-TR ADHD subtype, n (%)			.909^c^
Combined	173 (78.6)	174 (77.3)	
Hyperactive/Impulsive	1 (0.5)	1 (0.4)	
Inattentive	46 (20.9)	50 (22.2)	
Prior stimulant use, n (%)	83 (37.7)	79 (35.1)	.622^c^

a N includes all randomized patients.

b Analysis of variance (ANOVA) model: baseline = treatment (Type III sums of squares).

c Treatment comparison was analyzed using Fisher’s exact test.

Abbreviations: ADHD = attention-deficit/hyperactivity disorder; DSM-IV-TR = Diagnostic and Statistical Manual of Mental Disorders, Fourth Edition-Text Revision; N = total number of participants; n = number of participants in the specified category; SD = standard deviation.

### Study Period I (5 to 28 day screening period, Visit 1–2)

After providing privacy statement consent, potential participants completed an internet-based, nonidentifiable Adult ADHD Self-Report Scale-V1.1 (ASRS-V1.1) Screener. Those with an ASRS-V1.1 Screener raw score of ≥14 were given the option to electronically sign an additional online informed consent. Participants opting-in then answered additional inclusion/exclusion criteria questions via the study website. Those who met inclusion/exclusion criteria for the trial and who lived near an available investigative site were scheduled a live screening visit to confirm that the participant fully met all study inclusion/exclusion criteria. Site personnel obtained informed consent for the study from participants before conducting any study procedures. Participants underwent laboratory tests, an electrocardiogram (ECG) evaluation, a psychiatric evaluation, and a physical examination. Participants underwent a washout period if they had been taking medications excluded by the study protocol.

### Study Period II (12-week double-blind acute treatment phase, Visit 2–6)

Participants were assessed 2 weeks after Visit 2, then every 3 weeks until Visit 5, and then after 4 weeks, which was Visit 6 (end of the double-blind phase of the study). This study employed a double-blind, sham placebo lead-in period, which blinded investigators and participants to the initiation of active therapy. Participants were randomized 1∶1 to either the atomoxetine or placebo group at the site level in a double-blinded manner, determined by a computer-generated random sequence using an interactive voice response system. Investigators were informed that all participants might receive placebo during a lead-in period between Visit 2 and Visit 4, prior to randomization. Investigators did not know that the randomization of all participants actually occurred at Visit 2.

### Dosing

Atomoxetine-treated patients were dosed 40 mg/day (20 mg BID) for a minimum of 7 days. Following the last dose, the participants were dosed 80 mg/day (40 mg BID) for a minimum of 7 days. At or after Visit 5, the dose could be increased to the maximum of 100 mg/day (50 mg BID) based upon investigator judgment. One unscheduled dose change was allowed if needed for tolerability or safety. Participants unable to tolerate 40 mg/day were discontinued. Dosing was consistent with atomoxetine US label recommendations [18].

### Assessments

The BRIEF-A is a standardized measure consisting of 75 items within 9 nonoverlapping clinical scales that measure various aspects of adult executive functioning and self-regulation in the person’s everyday environment [19]. The BRIEF-A yields an overall score, the Global Executive Composite (GEC), which is a composite of 2 index scores: Behavioral Regulation Index (BRI) and Metacognitive Index (MI). The BRI is comprised of 4 subscales (Inhibit, Shift, Emotional Control, and Self-Monitor) and the MI is comprised of 5 subscales (Initiate, Working Memory, Plan/Organize, Task Monitor, and Organization of Materials). Patients rated behavior for each of the 75 items on a 3-point Likert scale (1 = behavior is never observed to 3 = behavior is often observed). GEC is a summary score that incorporates all of the clinical subscales of the BRIEF-A. Higher BRIEF-A scores indicate greater executive functioning impairment. Raw scores can be transformed (T-scores) based upon standardized population samples, with scores ≥65 having been considered to be clinically significant [20]. For assessment of treatment groups at baseline in these analyses, T-scores ≥60 were considered to be representative of clinical impairment. The percentage of patients with GEC, BRI, or MI T-scores ≥60 was measured at baseline.

The BRIEF-A also includes 3 built-in validity scales: Inconsistency, Infrequency, and Negativity, which check whether answers are provided in an inconsistent, atypical, or negative fashion, respectively, relative to normative and clinical samples.

Per the protocol, BRIEF-A assessments were collected at Visits 2 (baseline), 4 (5 weeks), and 6 (12 weeks, end of acute study period).

Safety analyses included treatment-emergent adverse events, weight, and vitals collected at every visit. Laboratory and ECG measures were collected study Visit 1 and 6. Although not the focus of this paper, safety findings were consistent with labeling, and have been described previously [16].

### Statistical Analyses

Sample size was selected to allow for more than 80% power on the primary efficacy measure (CAARS) rather than secondary measures. This was based upon results from previous atomoxetine trials. Assignment to treatment groups was determined by a computer-generated random sequence using an interactive voice response system at the site level in a double-blind fashion. Details have been described previously [16].

Age, weight, and height treatment group differences were assessed with analysis of variance (ANOVA), with baseline equal to treatment (Type III sums of squares). Other demographic treatment comparisons were assessed with Fisher’s exact test.

Mean changes from baseline to 12-week endpoint on the BRIEF-A were analyzed using a pre-specified analysis of covariance (ANCOVA) model with terms for baseline score, treatment, and investigator. Missing data were handled using last observation carried forward (LOCF) methodology. Hochberg’s p-value adjustment method was used to control for potential multiplicity issues when analyzing the four component scores of the Behavioral Regulation Index and the five component scores of the Metacognitive Index. Additionally, a post-hoc mixed model repeated measures (MMRM) analysis on the GEC score with terms for baseline score, treatment, investigator, visit, treatment-by-visit interaction (time-by-treatment), and baseline-score-by-visit interaction was performed as a sensitivity analysis. MMRM utilizes modeling based upon included terms to handle missing data. The LOCF and MMRM analyses are based upon raw scores rather than T-scores.

Effect sizes were calculated as mean change in atomoxetine group scores minus mean change in placebo group scores divided by the pooled standard deviation of the changes.

## Results

There were no statistically significant treatment group differences for any demographic parameter, including ADHD subtype, a majority of which was the combined type (Table 1). At baseline, there were no statistically significant differences in the percentage of patients with GEC, BRI, or MI T-scores ≥60. At baseline, 93.6% and 92.4% of the atomoxetine and placebo groups, respectively, had GEC T-scores ≥60, with 60 being 2 standard deviations away from the standardized mean (Table 2).

**Table 2:** BRIEF-A Baseline Subscale T-Scores.

	Atomoxetine (N = 219^a^)	Placebo (N = 224^a^)	
	n (%)	n (%)	*P*-value
GEC T-score ≥60 at baseline	205 (93.61)	207 (92.41)	.711
GEC T-score <60 at baseline	14 (6.39)	17 (7.59)	
BRI T-score ≥60 at baseline	162 (73.97)	166 (74.11)	1.0
BRI T-score <60 at baseline	57 (26.03)	58 (25.89)	
MI T-score ≥60 at baseline	204 (93.15)	212 (94.64)	.556
MI T-score <60 at baseline	15 (6.85)	12 (5.36)	

a n includes all randomized patients with a valid BRIEF-A score at baseline.

Abbreviations: BRI = Behavioral Regulation Index; GEC = Global Executive Composite; MI = Metacognitive Index; N = number of participants; n = number of participants in the specified category.

As published previously [16], the least squares mean change (± standard error) from baseline to end point in GEC score was statistically significantly greater for the atomoxetine group (−22.4±1.9) compared with the placebo group (−14.8±1.9; −12.37 to −2.78 95% confidence interval, *p* = .002). The mean ± standard deviation baseline and end point GEC scores for atomoxetine versus placebo were as follows: 155.0±21.5 to 135.2±28.4 (N = 161) versus 156.1±21.5 to 142.6±26.6 (N = 167). There was a statistically significantly greater decrease in mean change (± standard deviation) from baseline to last observation demonstrated in the atomoxetine group compared with the placebo group not only for the BRIEF-A overall GEC score but also the BRI and MI scores (Table 3).

**Table 3:** BRIEF-A Mean Change from Baseline to Last Observation for All Randomized Patients.

	Change to Last Observation		
	Atomoxetine (n = 161^a^)	Placebo (n = 167^a^)	
Index/Scale	Mean ± SD	Mean ± SD	*P*-value^b^
**Global Executive Composite**	−19.78±25.59	−13.41±21.76	.002
**Behavioral Regulation Index**	−7.22±11.77	−4.47±10.05	.007
Inhibit	−2.33±3.17	−1.44±2.71	.005
Shift	−1.32±2.69	−0.93±2.57	.090
Emotional Control	−1.67±4.97	−1.18±4.48	.219
Self-Monitor	−1.91±2.89	−0.92±2.51	.005
**Metacognitive Index**	−12.55±15.14	−8.93±13.35	.003
Initiate	−2.11±3.42	−1.71±3.11	.051
Working Memory	−3.08±3.65	−1.98±3.02	<.001
Plan/Organize	−3.22±4.52	−2.14±3.92	.010
Task Monitor	−1.96±2.46	−1.46±2.61	.023
Organization of Materials	−2.19±3.24	−1.66±3.09	.051

a n includes all randomized patients who also had a baseline score and at least one post-baseline score on the BRIEF-A scale.

b All *p*-values were statistically significant (*p*≤.05) except for Shift, Emotional Control, Initiative, and Organization of Materials; 9 component score p-values adjusted with Hochberg’s Method to control for potential multiplicity.

Abbreviations: n = number of participants; SD = standard deviation.

Based upon the difference between the effect of atomoxetine versus placebo on GEC scores (change from baseline to end point), the atomoxetine treatment effect size was −0.32, indicating a clinically relevant small to moderate effect [21]. The effect size is negative since lower scores are better than higher scores and the atomoxetine group had the lower (improved) scores.

While there were significant decreases in BRIEF-A scores for both atomoxetine- and placebo-treated patients, there was also a statistically significantly greater decrease in mean change from baseline to last observation observed in the atomoxetine compared with the placebo group for the following BRIEF-A subscales: Inhibit, Self-Monitor, Working Memory, Plan/Organize, and Task Monitor (Table 3). Mean changes in BRIEF-A Shift, Organization of Materials, Initiate, and Emotional Control subscale scores were not statistically significantly different between treatment groups.

Mean baseline validity scores for both treatment groups were similar, low, and within the acceptable range for scale validity. Changes from baseline in the BRIEF-A validity scale scores were not statistically significantly different between treatment groups: Inconsistency (*p* = .644), Infrequency (*p* = .097), and Negativity (*p* = .456). These data demonstrate patient scoring validity for the study.

Repeated measures analyses of GEC score change from baseline to each post-baseline visit (when BRIEF-A assessments were captured) showed statistically significant separation between the atomoxetine and placebo groups at Week 5 (Visit 4) and Week 12 (Visit 6) (Table 4). The overall treatment effect was also statistically significant (*p*<.001) for BRIEF-A GEC score change from baseline using repeated measures analysis.

**Table 4:** BRIEF-A Mean Change from Baseline to Postbaseline Visits for All Randomized Patients – Repeated Measures Analysis for Global Executive Composite Scores.

Week	Treatment	n^a^	LSMean Change ± SE	Difference	*P*-value	95% CI
5	Atomoxetine	157	−18.02±1.69	−7.11	.002	−11.49 to −2.73
	Placebo	166	−10.91±1.67			
12	Atomoxetine	115	−22.53±2.07	−7.78	.005	−13.19 to −2.36
	Placebo	128	−14.75±2.00			

a n includes all randomized patients who also had a BRIEF-A baseline score and a postbaseline score at Visit 4 (Week 5) and/or Visit 6 (Week 12), as applicable.

Abbreviations: CI = confidence interval; LS = least squares; n = number of participants; SE = standard error.

## Discussion

In the currently described ADHD study, statistically significantly greater mean reductions were observed for the atomoxetine- versus placebo-treated patients for the BRIEF-A Global Executive Composite (GEC), Behavioral Regulation Index (BRI), and Metacognitive Index (MI) scores, as well as the Inhibit, Self-Monitor, Working Memory, Plan/Organize, and Task Monitor subscale scores. Thus, greater improvement in executive function was observed in young adults with ADHD in the atomoxetine versus placebo group as measured by changes in the BRIEF-A scales.

ADHD is known to be a neurological behavioral disorder. Multiple studies have shown that there are size, structural, and signaling differences in several regions and structures in the brains of patients with ADHD compared with normal controls. These differences are thought to be connected to the development of ADHD and its associated symptoms [7], [8], [9], [10], [11], [22]. One area of focus for ADHD is the prefrontal cortex that is involved in attention and executive functioning; more specifically, the dorsal anterior cingulate cortex and dorsolateral prefrontal cortex involved in inattention, the prefrontal motor cortex involved in hyperactivity, and the orbital frontal cortex involved in impulsivity [11]. An important function of catecholamines such as norepinephrine in these brain areas may be to facilitate or inhibit behavior [7], [12], [13], and medications that modulate this signaling may have effects on executive functioning [23]. The precise mechanism by which atomoxetine produces its therapeutic effects in ADHD is unknown, but it is thought to be related to selective inhibition of the pre-synaptic norepinephrine transporter, as determined in ex vivo uptake and neurotransmitter depletion studies [24]. Thus, atomoxetine may affect executive functioning via its actions on norepinephrine levels in the central nervous system. Additionally, while atomoxetine is selective for norepinephrine transporters, microdialysis studies suggest that atomoxetine may selectively increase dopamine levels in the prefrontal cortex, via the norepinephrine transporter or perhaps via a secondary signaling mechanism [23]. This could also play a role in signaling that affects executive function.

Interpretation of the currently presented BRIEF-A results should be tempered by the absence of neuropsychological assessments of executive function. However, neuropsychological tests have not been shown to reliably predict functional impairment in adult ADHD, while clinical ratings such as the BRIEF have been successful in this regard [25], [26]. Moreover, Biederman and colleagues [27] found only a modest overlap between psychometric and self-reported measures of executive function impairment among ADHD patients. Neuropsychological testing was largely found to identify patients with lower IQ and achievement testing, while behavioral questionnaire assessment chiefly identified patients with higher levels of ADHD symptoms, psychiatric comorbidity, and interpersonal deficits. As measured by the BRIEF-A at the end of 5 weeks and at the end of 12 weeks of treatment, atomoxetine treatment significantly improved executive functioning in young adults with ADHD compared with placebo. This is reflected in changes in the overall composite score, two index scores, and scores for seven of the nine BRIEF-A subscales (all but emotion control and organization). Over 90% of patients in this study had a GEC T-score ≥60 which shows an impairment of greater than 2 standard deviations from the mean, suggesting an ADHD population with deficits in executive functioning. Additionally, results from the three validity scales built into the BRIEF-A suggest valid self-reporting when completing the BRIEF-A assessment and provide strength to the merit of the atomoxetine versus placebo results. These results demonstrate that atomoxetine reduces symptoms of ADHD believed to be associated with deficits in executive functioning in young adults compared with placebo. These results are also consistent with findings from another recent double-blind, placebo-controlled trial of adults with ADHD, where atomoxetine was associated with significant improvement in executive function as measured by the Brown Attention-Deficit Disorder Scale (BADDS), which measures executive functioning across 5 clusters of daily activity functioning [4]. Statistically significant differences in score mean change over a 6-month period were observed for BADDS total score and all 5 cluster scores.

Similar findings (score reductions across all BRIEF-A scales) to those presented here were observed in a recent randomized, double-blind, placebo-controlled, adult study evaluating the efficacy of lisdexamfetamine dimesylate (prodrug of dextroamphetamine) for executive function deficits in adults with ADHD as measured by the self-reported BRIEF-A [28]. Amphetamines are believed to block the reuptake of norepinephrine and dopamine into the presynaptic neuron and increase the release of these monoamines into the extraneuronal space [29]. This could be a mechanism by which lisdexamfetamine dimesylate affected executive functioning. Other stimulant ADHD medications that affect norepinephrine and dopamine levels, such as methylphenidate, have also shown efficacy for improving deficits in some executive functioning categories but not others [30], [31]. Additionally, some aspects of executive functioning in patients with neurocognitive deficits caused by diseases other than ADHD have been shown to improve with administration of stimulants [32], [33].

Of note in the current study is that there were no statistically significant treatment group differences for the Shift (*p* = .090), Organization of Materials (*p* = .051), Initiate (*p* = .051), and the Emotional Control (*p* = .219) subscale scores, with *p*≤.05 denoting significance after Hochberg’s p-value adjustment was used to control for potential multiplicity. Similar to this study, in the lisdexamfetamine study, the effect of lisdexamfetamine on BRIEF-A subscale scores was least pronounced for the emotional control score compared with any of the other subscale scores [28]. This may indicate that emotional regulation may track separately from other executive function domains for ADHD patients. Low baseline scores for emotional control compared to the other index groups could have been a factor in the results for these studies. However, in comparison, in the atomoxetine study using the BADDS to assess executive functioning, the emotion domain had the lowest baseline BADDS cluster score and the lowest mean change from baseline score; but, in this case, the effect of atomoxetine on reducing the emotion cluster score was statistically significantly greater than placebo [4].

Potential limitations of the current results are that limited adjustments for multiple comparisons were made that included an overall gatekeeper analysis [16] and use of Hochberg’s p-value adjustment for analysis of BRIEF-A component scores. Although not commonly used in adult ADHD trials [34], there was an absence of formal informant ADHD ratings. There was also an overrepresentation of white participants relative to African Americans and Hispanics compared with the overall US demographics. Additionally, this study had a higher-than-expected drop out rate, which could have reduced the power of the study. The BRIEF-A assessments were only conducted at baseline, Week 5, and Week 12. For the BRIEF-A analyses, only those patients with both a baseline and post-baseline measurement were included. The drop-out rate and the large window before the first assessment accounts for the decrease in patient number between baseline demographics and the BRIEF-A analyses.

These data, combined with the fact that the BRIEF-A subscales cover 75 items within 9 nonoverlapping clinical scales measuring various aspects of executive functioning (inhibition, shifting, emotion, self-monitoring, initiation, memory, planning/organizing, monitoring, and organization), support the utility of the BRIEF-A scale in measuring responses to atomoxetine of young adults with ADHD. Moreover, these results provide evidence that executive functioning impairments exist beyond the core symptoms of ADHD noted as diagnostic criteria in the DSM-IV-TR. The BRIEF-A has advantages as a behavioral rather than neuropsychological assessment of executive functioning in that it relies on ratings of real-world life activities [26]. Use of an executive function assessment such as the BRIEF-A may be an important tool for clinicians in diagnosing and monitoring ADHD symptomology relevant to patient real-world functioning outcomes and/or at least for managing a cognitive comorbidity often associated with ADHD [35].

## Supporting Information

Checklist S1 **CONSORT checklist of information to include when reporting a randomized trial.**
(DOCX)

Protocol S1 **A double-blind study of atomoxetine hydrochloride versus placebo for the treatment of ADHD in young adults with an assessment of associated functional outcomes.**
(DOCX)
