# Supplementary material for: Atomoxetine Effects on Executive Function as Measured by the BRIEF-A in Young Adults with ADHD: A Randomized, Double-Blind, Placebo-Controlled Study
Source: PLoS One. 2014 Aug 22;9(8):e104175. doi: 10.1371/journal.pone.0104175 (PMC4141744; doi:10.1371/journal.pone.0104175)
Supplement: Protocol S1 — A double-blind study of atomoxetine hydrochloride versus placebo for the treatment of ADHD in young adults with an assessment of associated functional outcomes. (DOCX) [file pone.0104175.s002.docx]

**PROTOCOL S1**

**1. Protocol B4Z-US-LYDZ (b)**

**A Double-Blind Study of Atomoxetine Hydrochloride versus Placebo for the Treatment of ADHD in Young Adults with an Assessment of Associated Functional Outcomes**

**Confidential Information**

The information contained in this protocol is confidential and is intended for the use of clinical investigators. It is the property of Eli Lilly and Company or its subsidiaries and should not be copied by or distributed to persons not involved in the clinical investigation of Atomoxetine hydrochloride (LY139603), unless such persons are bound by a confidentiality agreement with Eli Lilly and Company or

its subsidiaries.

Atomoxetine Hydrochloride (LY139603)

Study LYDZ(b) is a Phase IV, multi-center double-blind, placebo-controlled trial

of atomoxetine treatment in young adults with ADHD. Patients will be treated for

12 weeks in the double-blind phase with an option to enter a 12-week open-label extension period.

Eli Lilly and Company

Indianapolis, Indiana USA 46285

Protocol Approved by Lilly: 17 November 2006

Protocol Amendment (a) Approved by Lilly: 25 April 2007

Protocol Amendment (b) Approved by Lilly: 17 December 2008

**2. Synopsis**

**Study Rationale**

ADHD in young adulthood is associated with significant functional impairments, thus, treating young adults with ADHD may prove critically important in alleviating or lessening these. The objective of Study LYDZ(b) is to prospectively evaluate atomoxetine’s efficacy in treating ADHD symptoms and atomoxetine’s effect on functional outcomes in the young adult patient population.

**Clinical Protocol Synopsis: Study B4Z-US-LYDZ(b)**

| **Name of Investigational Product:** Atomoxetine hydrochloride (LY139603) | |
| --- | --- |
| **Title of Study:** A Double-Blind Study of Atomoxetine Hydrochloride versus Placebo for the Treatment of ADHD in Young Adults with an Assessment of Associated Functional Outcomes | |
| **Number of Planned Patients/Subjects:**  Entered: 550  Enrolled/Randomized: 446  Completed: 117 | **Phase of Development:** IV |
| **Length of Study:** 19 Months  Planned first patient visit: 16 August 2007 Planned last patient visit: 10 March 2009 | |
| **Objectives:**  **Primary Objective:** The primary objective of this study is to compare the efficacy of atomoxetine at  40-100 mg/day dosed BID and placebo on the reduction of ADHD symptoms after 12 weeks of double- blind treatment as measured by the mean change in the Conners’ Adult ADHD Rating Scale- Investigator Rated: Screening Version (CAARS-Inv:SV) total ADHD symptom score in young adults ages 18 to 30 who meet criteria for ADHD as defined by DSM-IV-TR.  **Secondary Objectives:** The secondary objectives of the study are:  • To assess the efficacy of atomoxetine therapy compared with placebo on the total score of the  Adult ADHD Quality of Life -29 (AAQOL-29).  • To assess the efficacy of atomoxetine therapy compared with placebo on the subscales of the AAQOL-29 in the following order: Relationship; Life Productivity; Psychological Health; and Life Outlook.  • To test the hypothesis that atomoxetine improves symptoms of ADHD compared to placebo after  12 weeks of treatment on the mean change from baseline to endpoint as measured by:  -Clinical Global Impression-ADHD-Severity (CGI-ADHD-S)  -CAARS Self Report (CAARS-S:SV)  -Patient Global Impression – Improvement (PGI-I)  • Montgomery Asberg Depression Rating Scale (MADRS): To assess the effect of atomoxetine in treating symptoms of depression in adults with ADHD as measured by the mean change in the total score after 12 weeks of treatment.  • Beck Anxiety Inventory (BAI): To assess the effect of atomoxetine in treating symptoms of anxiety in adults with ADHD as measured by the mean change in the total score after 12 weeks of treatment.  • To assess the correlation between the improvements on the AAQOL-29 Total Score with symptom reduction as measured by improvement on the CAARS-Inv:SV total ADHD symptom score. | |
| **Study Design:** Study B4Z-US-LYDZ(b) is a multi-center, 12-week, randomized, placebo-controlled, double-blind, trial with 3 study periods for the evaluation of safety and efficacy between atomoxetine and placebo in approximately 440 adult outpatients (age 18 to 30) with ADHD at approximately 35 sites in the United States and Puerto Rico. The protocol design contains a community sample (CS), and non- interventional health outcomes data will be collected from CS participant via web-based self-reporting done through the study website. | |
| **Diagnosis and Main Criteria for Inclusion and Exclusions:** Male or female outpatients 18 to 30 years of age meeting *Diagnostic and Statistical Manual of Mental Disorder, Fourth Edition Text Revision* (DSM-IV-TR) criteria for Attention-Deficit/Hyperactivity Disorders (ADHD) as assessed by the ADHD Clinician Diagnostic Scale (ACDS). | |

| **Test Product, Dosage and Mode of Administration:** In the double-blind phase, atomoxetine 40-  100 mg/day given as 20-, 25-, or 40-mg capsules orally, twice daily (BID). In the open-label extension period, atomoxetine 36-100 mg/day given as 18-, 40-, or 60-mg capsules orally, once daily (QD) or BID. |
| --- |
| **Planned Duration of Treatment:** Patients will be treated for 12 weeks in the double-blind phase with an option to enter a 12-week open-label extension period.  screening period (Study Period I): 5 to 28 days treatment period (Study Period II): 12 weeks  optional open-label extension period (Study Period III): 12 weeks |
| **Reference Therapy, Dose and Mode of Administration:** Placebo in capsule form given orally BID. |
| **Criteria for Evaluation:**  Primary Efficacy Measure:  Conners’ Adult ADHD Rating Scale-Investigator Rated: Screening Version (CAARS-Inv:SV) Secondary Efficacy Measures:  Adult ADHD Quality of Life (AAQOL-29)  Clinical Global Impression-ADHD-Severity (CGI-ADHD-S)  Conners’ Adult Attention-Deficit/Hyperactivity Disorder Rating Scale–Self Report: Screening Version- Administered (CAARS-S:SV)  Patient Global Impression-Improvement (PGI-I) Montgomery Asberg Depression Rating Scale (MADRS) Columbia Suicide-Severity Rating Scale:  Beck Anxiety Inventory (BAI)  Secondary Functional Outcome Measures:  Habits Timeline Followback (TLFB): Alcohol, Nicotine, Caffeine, Marijuana, and Drugs  Fagerstrom Test for Nicotine Dependence (FTND) Social Adaptation Self-Evaluation Scale (SASS) Driving Behavior Survey (Self-Report)  Driving Behavior Survey (Other-Report)  Behavior Rating Inventory of Executive Function-Adult Version (BRIEF-A) Epworth Sleepiness Scale (ESS)  Safety: Safety will be assessed by summarizing and analyzing adverse events, vital signs, weight, electrocardiograms (ECG), and concomitant medications use. |

**Statistical Methods:**

Statistical:

The primary summary measure, the CAARS-Inv:SV total ADHD symptom score as well as the Inattention subscale score, and Hyperactivity-Impulsivity subscale score, will be analyzed using a restricted maximum likelihood (REML)-based, mixed model repeated measures (MMRM) technique. The model will include the fixed, categorical effects of treatment, investigator, visit, and treatment-by-

visit interaction as well as the continuous, fixed covariates of baseline (last of scores at Visit 1 and Visit 2) score and baseline score-by-visit interaction. Secondary evidence of efficacy from the primary analysis will be based on the main effect of treatment and the treatment-by-visit interaction terms from the

MMRM analysis. This measure will be summarized separately during Study Period III. All statistical tests will be two-sided and will be conducted at the 5% significance level.

As supporting evidence, the CAARS-Inv:SV total ADHD symptom score as well as the Inattention subscale score, and Hyperactivity-Impulsivity subscale score, will be analyzed using a last observation carried forward (LOCF) analysis. Change from baseline to LOCF endpoint will be compared across treatments using an ANCOVA model with terms for baseline, treatment, and investigator.

The first gated secondary objective is to assess the efficacy of atomoxetine therapy compared with placebo on the total score of the Adult ADHD Quality of Life -29 (AAQOL-29) using a treatment contrast from the repeated measures mixed effects model at the final post baseline visit. The repeated measures model will include the same terms as that used for the primary efficacy measure. This measure will be summarized separately during Study Period III.

The next gated secondary objective is to assess the efficacy of atomoxetine therapy compared with placebo on the subscales of the AAQOL-29 in the following order: Relationship; Life Productivity; Psychological Health; and Life Outlook using a treatment contrast from the repeated measures mixed effects model at the final post baseline visit. The repeated measures model will include the same terms as that used for the primary efficacy measure. These subscale measures will be summarized separately during Study Period III.

Approximately 440 patients will be randomized in a 1:1 ratio between atomoxetine and placebo

(220 patients per group). This sample size has been selected to allow for more than 80% power at the end of the study, assuming a 68% retention rate over 12 weeks. Safety analysis will include all randomized patients who take at least one dose of study drug. The incidence of treatment-emergent adverse events (TEAE) will be summarized by the following subgroups; investigational site, prior-stimulant-use strata, ADHD subtype, gender, and age. No interim analyses are planned for this study.

**3. Table of Contents**

**A Double-Blind Study of Atomoxetine Hydrochloride versus Placebo for the Treatment of ADHD in Young Adults with an Assessment of Associated Functional Outcomes**

**Section Page**

1. Protocol B4Z-US-LYDZ (b) A Double-Blind Study of Atomoxetine Hydrochloride versus Placebo for the Treatment of ADHD in Young Adults with an Assessment of Associated

Functional Outcomes........................................................................... 1

2. Synopsis.............................................................................................. 2

3. Table of Contents ................................................................................ 6

4. Abbreviations and Definitions ........................................................... 11

5. Introduction....................................................................................... 14

6. Objectives ......................................................................................... 16

6.1. Primary Objective ........................................................................ 16

6.2. Secondary Objectives ................................................................... 16

6.2.1. Gated Secondary Objectives ................................................. 16

6.2.2. Additional Secondary Objectives.......................................... 16

6.2.3. Secondary Functional Outcome Objectives........................... 17

7. Investigational Plan ........................................................................... 18

7.1. Summary of Study Design ............................................................ 18

7.2. 23Discussion of Design and Control............................................. 23

8. Study Population ............................................................................... 24

8.1. Inclusion Criteria .......................................................................... 24

8.1.1. Disease Diagnostic Criteria .................................................. 25

8.2. Exclusion Criteria......................................................................... 25

8.2.1. Rationale for Exclusion of Certain Study Candidates............ 28

8.3. Discontinuations........................................................................... 28

8.3.1. Discontinuation of Patients ................................................... 28

8.3.2. Discontinuation of Study Sites.............................................. 29

8.3.3. Discontinuation of the Study ................................................ 29

9. Treatment .......................................................................................... 30

9.1. Treatments Administered.............................................................. 30

9.1.1. Study Period II ..................................................................... 30

9.1.2. Study Period III .................................................................... 31

9.2. Materials and Supplies.................................................................. 31

9.3. Method of Assignment to Treatment............................................. 32

9.4. Rationale for Selection of Doses in the Study ............................... 32

9.5. Selection and Timing of Doses ..................................................... 33

9.5.1. Special Treatment Considerations......................................... 33

9.6. Blinding ....................................................................................... 33

9.7. Concomitant Therapy ................................................................... 34

9.8. Treatment Compliance ................................................................. 34

10. Efficacy, Health Outcome/Quality of Life Measures, and

Safety Evaluations, and Appropriateness of Measurements................ 35

10.1. Efficacy Measures ........................................................................ 35

10.1.1. Primary Efficacy Measure .................................................... 35

10.1.1.1. Conners’ Adult ADHD Rating Scale-Investigator

Rated: Screening Version (CAARS-Inv:SV) ................... 35

10.1.2. Secondary Efficacy Measures ............................................... 35

10.1.2.1. Adult ADHD Quality of Life (AAQOL-29) .................... 35

10.1.2.2. Clinical Global Impression—ADHD-Severity

(CGI-ADHD-S) .............................................................. 36

10.1.2.3. Conners’ Adult Attention-Deficit/Hyperactivity

Disorder Rating Scale–Self Report−Screening

Version-Administered (CAARS-S:SV*)* ........................... 36

10.1.2.4. Patient Global Impression-Improvement (PGI-I)............. 36

10.1.2.5. Montgomery Asberg Depression Rating Scale

(MADRS) ....................................................................... 36

10.1.2.5.1. Columbia Suicide-Severity Rating Scale: .................. 36

10.1.2.6. Beck Anxiety Inventory (BAI) ........................................ 37

10.2. Secondary Functional Outcome Measures .................................... 37

10.2.2. Habits Timeline Followback (TLFB): Alcohol,

Nicotine, Caffeine, Marijuana, and Drugs............................. 37

10.2.3. Fagerstrom Test for Nicotine Dependence (FTND) .............. 37

10.2.4. Social Adaptation Self-Evaluation Scale (SASS) .................. 37

10.2.5. Driving Behavior Survey (Self-Report) ................................ 37

10.2.6. Driving Behavior Survey (Other-Report).............................. 38

10.2.7. Behavior Rating Inventory of Executive Function-

Adult Version (BRIEF-A) .................................................... 38

10.2.8. Epworth Sleepiness Scale (ESS) ........................................... 38

10.3. Safety Evaluations ........................................................................ 39

10.3.1. Adverse Events .................................................................... 39

10.3.1.1. Serious Adverse Events (SAEs) ...................................... 40

10.3.2. Other Safety Measures ......................................................... 41

10.3.3. Safety Monitoring ................................................................ 41

10.3.4. Complaint Handling ............................................................. 41

10.4. Sample Collection and Testing ..................................................... 42

10.4.1. Samples for Standard Laboratory Testing ............................. 42

10.5. Appropriateness of Measurements ................................................ 43

11. Data Quality Assurance ..................................................................... 44

11.1. Direct Data Entry and Computerized Systems .............................. 44

12. Sample Size and Statistical Methods ................................................. 46

12.1. Determination of Sample Size ...................................................... 46

12.2. Statistical and Analytical Plans ..................................................... 47

12.2.1. General Considerations ........................................................ 47

12.2.1.1. Analysis Populations....................................................... 47

12.2.1.2. Adjustment for Multiplicity ............................................ 48

12.2.2. Patient Disposition ............................................................... 48

12.2.3. Patient Characteristics .......................................................... 48

12.2.4. Concomitant Therapy ........................................................... 48

12.2.5. Treatment Compliance ......................................................... 49

12.2.6. Primary Outcome and Methodology ..................................... 49

12.2.7. Secondary Analyses of the Primary Outcome ....................... 49

12.2.8. Gated Secondary Efficacy Analyses ..................................... 49

12.2.8.1. Secondary Efficacy Analyses .......................................... 50

12.2.9. Safety Analyses .................................................................... 52

12.2.10. Subgroup Analyses............................................................... 53

12.2.11. Interim Analyses .................................................................. 54

13. Informed Consent, Ethical Review, and Regulatory

Considerations................................................................................... 55

13.1. Informed Consent ......................................................................... 55

13.2. Ethical Review ............................................................................. 55

13.3. Regulatory Considerations............................................................ 56

13.3.1. Investigator Information ....................................................... 56

13.3.2. Protocol Signatures .............................................................. 57

13.3.3. Final Report Signature.......................................................... 57

14. References......................................................................................... 58

Introduction and Rationale ...................................................................... 75

Objectives and the Corresponding Measures ........................................... 75

Study Design and Brief Discussion of the Design ................................... 76

Power Analysis and Statistical Methods for Analyses ............................. 77

Power analysis ..................................................................................... 77

Statistical Analysis .............................................................................. 77

Schedule of Events ................................................................................. 78

References .............................................................................................. 79

**Table of Contents (concluded)**

**List of Protocol Attachments**

Protocol Attachment LYDZ(b).1.

Study Schedule

Protocol Attachment LYDZ(b).2.

Clinical Laboratory Tests

Protocol Attachment LYDZ(b).3.

Concomitant Medication Table

Protocol Attachment LYDZ(b).4.

Adult Electrocardiogram Alert Criteria

Protocol Attachment LYDZ(b).5.

Community Sample

**4. Abbreviations and Definitions**

**AAQOL-29** Adult ADHD Quality of Life-29

**ACDS v1.2** Adult ADHD Clinician Diagnostic Scale version 1.2

**ADHD** Attention-Deficit/Hyperactivity Disorder

**ASRS** Adult ADHD Symptom Rating Scale v1.1

**Active Treatment or Active Study Drug**

Indicates that the patient is receiving atomoxetine.

**Adverse Event (AE)** Any untoward medical occurrence in a patient or clinical investigation subject administered a pharmaceutical product and which does not necessarily have a causal relationship with this treatment. An adverse event can therefore be any unfavorable and unintended sign (including an abnormal laboratory finding), symptom, or disease temporally associated with the use of a medicinal (investigational) product, whether or not related to the medicinal (investigational) product.

**Audit** A systematic and independent examination of the trial-related activities and documents to determine whether the evaluated trial-related activities were conducted, and the data were recorded, analyzed, and accurately reported according to the protocol, applicable standard operating procedures (SOPs), good clinical practice (GCP), and the applicable regulatory requirement(s).

**Blinding/Masking** A procedure in which one or more parties to the trial are kept unaware of the treatment assignment(s). Double-blinding usually refers to the subject(s), investigator(s), monitor(s), and in some cases, select sponsor personnel being unaware of the treatment assignment(s).

**BRIEF-A** Behavior Rating Inventory of Executive Function – Adult Version

**CAARS-Inv:SV** Conners’ Adult ADHD Rating Scale-Investigator Rated: Screening Version

**CAARS-S:SV** Conners’ Adult ADHD Rating Scale-Self Report: Screening Version

**CGI-ADHD-S** Clinical Global Impression: ADHD-Severity

**Complaint** A complaint is any written, electronic, or oral communication that alleges deficiencies related to the identity, quality, purity, durability, reliability, safety

or effectiveness, or performance of a drug or drug delivery system.

**Compliance** Adherence to all the trial-related requirements, good clinical practice (GCP)

requirements, and the applicable regulatory requirements.

**CS** Community Sample – The community sample is a superset of subjects (who do not qualify or qualify but choose not to participate in the randomized research clinical trial (RCT) or cannot participate because an investigator site is not within their geography) from which we will obtain non-interventional health outcomes data via web-based self-reporting.

**DSM-IV™-TR** Diagnostic and Statistical Manual of Mental Disorders, Fourth Edition- Text

Revision

**ECG** Electrocardiogram

**ESS** Epworth Sleepiness Scale

**eCRF** Electronic case report form (sometimes referred to as an electronic clinical report form). An electronic form for recording study participants’ data during a clinical study, as required by the protocol.

**End of Study (Trial)** The end of the study is defined as the last visit of the last patient.

**Enroll** See Study Entry Terms

**Enter** See Study Entry Terms

**ePRO** Electronic Patient Reported Outcomes: Patient- or observer-rated scales that are collected via an electronic data capture system.

**FTND** Fagerstrom Test for Nicotine Dependence

**Full Analysis Set**

**(FAS)**

The set of patients that is as close as possible to the ideal implied by the intention-to-treat principle (all randomized patients identified by assigned treatment group, even if they deviate from the protocol or course of treatment).

**Interim Analysis** Any analysis intended to compare treatment groups at any time prior to the formal completion of a trial.

**Intention to Treat**

**(ITT)**

The principle that asserts that the effect of a treatment policy can be best assessed by evaluating on the basis of the intention to treat a subject (that is, the planned treatment regimen) rather than the actual treatment given. It has the consequence that subjects allocated to a treatment group should be followed up, assessed and analyzed as members of that group irrespective of their

compliance to the planned course of treatment.

**Investigator** A person responsible for the conduct of the clinical trial at a trial site. If a trial is conducted by a team of individuals at a trial site, the investigator is the responsible leader of the team and may be called the principal investigator.

**IRB/ERB** Institutional review board/ethical review board: A board or committee (institutional, regional, or national) composed of medical professional and non- medical members whose responsibility is to verify that the safety, welfare, and human rights of the subjects participating in a clinical trial are protected.

**MADRS** Montgomery-Asberg Depression Rating Scale

**MDD** Major depressive disorder

**Patient** A subject with a defined disease.

Those individuals participating in the Research Clinical Trial will be referred to as patients.

**PGI-I** Patient Global Impression - Improvement

**PHQ-9** Patient Health Questionnaire

**RCT** Research Clinical Trial

**SASS** Social Adaptation Self-Evaluation Scale

**SAE** Serious Adverse Event

**SCID-RV** Structured Clinical Interview for DSM-IV™-TR Axis I Disorders- Research

Version

**Study Entry Terms** Screen

The act of determining if an individual meets minimum requirements to become part of a pool of potential candidates for participation in a clinical trial. In this study, pre-screening occurs via questions answered on a study website. Eligible patients may then be further screened (for example, diagnostic psychological tests, blood draws) at the investigator site level. A privacy statement and/or informed consent is obtained prior to individuals answering any questions to determine eligibility for the trial.

Enter/Enrolled

Entered patients are those patients who meet pre-screening requirements (from the web-based screening phase) and are deemed eligible to proceed with further evaluation to determine eligibility for the Research Clinical Trial at an investigator site. Entered patients will have at least a Visit 1 recorded.

Enrolled patients are those patients who meet all of the inclusion criteria and none of the exclusion criteria, agree to continue participation in the study, and complete the Visit 2. These patients are considered as “enrolled in the study” and will receive study medication at the end of Visit 2.

**Subject** An individual who is or becomes a participant in clinical research, either as a recipient of the test article or as a control. A subject may be either a healthy human or a patient.

Those individuals participating in screening and those who are in the

Community Sample of the trial will be referred to as subjects.

**TLFB** Habits Timeline Followback (TLFB): Alcohol, Nicotine, Caffeine, Marijuana, and Drugs

**Treatment- Emergent Adverse Event (TEAE)**

Any untoward medical occurrence that either occurs or worsens at any time after treatment baseline and which does not necessarily have to have a causal relationship with this treatment (also called treatment-emergent signs and symptoms [TESS]).

**A Double-Blind Study of Atomoxetine Hydrochloride versus Placebo for the Treatment of ADHD in Young Adults with an Assessment of Associated Functional Outcomes**

**5. Introduction**

Atomoxetine hydrochloride1 (hereafter referred to as atomoxetine), a potent and highly selective inhibitor of the presynaptic norepinephrine transporter, is approved by the FDA as a treatment for Attention-Deficit/Hyperactivity Disorder (ADHD) in children, adolescents, and adults, as defined by the Diagnostic and Statistical Manual of Mental Disorders, Fourth Edition-Text Revision (DSM-IV-TR™); (APA [American Psychiatric Association] 2000). Atomoxetine was approved for use in adults with ADHD based on two pivotal trials: B4Z-US-LYAA and B4Z-US-LYAO (Michelson et al. 2003).

ADHD in young adulthood can create life-long devastating functional outcomes, thus, treating young adults with ADHD is critically important. Sixty percent of children and adolescents who have ADHD continue to have symptoms of ADHD into adulthood (Hechtman 1992; Barkley 1998). Impairment due to ADHD can have potentially long- lasting negative effects in young adults as they transition into adulthood. Decisions made by young adults as they move away from home, go to college, and start careers affect the trajectories of their lives and can having sustaining impact. Furthermore, as children and adolescents with ADHD transition into adulthood, they are more likely to develop comorbid psychopathology and engage in high risk behaviors than their non-ADHD counterparts (Biederman et al. 2006). In a 10-year prospective study in male youths with ADHD, subjects reported higher rates of anti-social, addictive, mood, and anxiety disorders compared to control subjects (Biederman et al. 2006). If left untreated, ADHD impairment puts this patient population at greater risk for negative life-long

consequences. For example, young adults with ADHD are more likely to have problems

sustaining long-term intimate relationships and twice as likely to be divorced or separated

(Biederman et al. 1993). Further, studies of driving behaviors in young adults with ADHD showed greater risks of speeding, traffic violation citations, and a tendency for more auto crashes than counterparts without ADHD. (Barkley et al. 1996). Additionally, the propensity for addictive behaviors is greater in adults with ADHD in that they are more likely to be smokers and are more likely to have alcohol and substance abuse comorbidities (Biederman et al. 1993; Pomerleau et al. 1995).

1 Atomoxetine or atomoxetine hydrochloride was formerly known as tomoxetine or tomoxetine hydrochloride. In agreement with the USAN Council (United States Adopted Names Council), the generic name was changed to minimize the possibility of confusion with tomoxifen, which is often used in the treatment for breast cancer.

The treatment of ADHD in the young adult is critical. Many therapies besides atomoxetine pose potential risks that are heightened in this particular demographic population. Therapies currently available for the treatment of ADHD, other than atomoxetine, include prescription stimulants such as methylphenidates, dextroamphetamine, and mixed amphetamines salts. However, these types of medications carry a risk for abuse and diversion (sell, trade, or give away), particularly for college-aged students and young adults. Varying reports of non-medical use of methylphenidates among college students suggests a lifetime use ranging from 3% to

16% and use within the past year of 5.7% (McCabe et al. 2005). Further, a study

sponsored by Eli Lilly reported lifetime non-medical use of long-acting stimulants (approximately 2.1%) (Novak et al. 2006). When examining the rate of diversion of prescribed stimulants among undergraduate students prescribed stimulants, 54% reported being approached to divert their medication (McCabe et al. 2006).

Atomoxetine is not subject to the problems of diversion and abuse associated with stimulants. Furthermore, atomoxetine may offer a treatment for ADHD and its negative functional outcomes in this specific population that is particularly effective. To date, this potential effectiveness has only been explored retrospectively. The question of which patients will most likely respond to a trial of atomoxetine is one that is very pertinent to prescribers. Many physicians believe that atomoxetine response is, in general, less robust than they would expect from some other alternative stimulant medications for ADHD. However, post hoc analyses of previous Lilly trials (B4Z-US-LYBV, B4Z-MC-LYAA, B4Z-MC-LYAO, and B4Z-US-LYCU) suggest that young adults respond robustly to atomoxetine. There is evidence to suggest that young adults may represent a

demographic group whose response to atomoxetine is more robust than physicians

typically expect; however, these findings have been replicated only in a post hoc fashion and have not been confirmed in a prospective trial. The objective of Study LYDZ(b) is to prospectively evaluate atomoxetine’s efficacy in treating ADHD symptoms and atomoxetine’s effect on functional outcomes in the young adult patient population.

In addition to the aims of the randomized clinical trial portion of Study LYDZ(b) discussed earlier, this study incorporates a community sample which is a superset of subjects who do not qualify or qualify but choose not to participate in the randomized research clinical trial (RCT) or qualify but cannot participate because an investigator site is not within their geography. From this superset of subjects, we will obtain non-

interventional health outcomes data via web-based self reporting to assess the

associations between ADHD symptom complexity and severity on functional outcomes for young adults with ADHD in this community sample. Results from the community sample will be reported separately from the main protocol study report. The Protocol Attachment LYDZ(b).5 further describes the community sample.

More detailed information about the known benefits and risks of atomoxetine hydrochloride may be found in the Investigator's Brochure (IB).

**6. Objectives**

**6.1. Primary Objective**

The primary objective is to compare the efficacy of atomoxetine at 40 - 100 mg/day dosed BID and placebo on the reduction of ADHD symptoms after 12 weeks of double- blind treatment as measured by the mean change in the Conners’ Adult ADHD Rating Scale- Investigator Rated: Screening Version (CAARS-Inv:SV) total ADHD symptom score in young adults ages 18 to 30 who meet criteria for ADHD as defined by DSM-IV- TR.

**6.2. Secondary Objectives**

***6.2.1. Gated Secondary Objectives***

The following two secondary objectives will be tested using the Gatekeeper strategy (Westfall 2001). Atomoxetine and placebo will be compared in a stepwise fashion in order as indicated below. Testing will stop when a measure fails to show statistical significance favoring atomoxetine (p<0.05) based on a 2-sided test of significance.

• To assess the efficacy of atomoxetine therapy compared with placebo on the total score of the Adult ADHD Quality of Life -29 (AAQOL-29).

• To assess the efficacy of atomoxetine therapy compared with placebo on the subscales of the AAQOL-29 in the following order: Relationship; Life Productivity; Psychological Health; and Life Outlook.

***6.2.2. Additional Secondary Objectives***

• To test the hypothesis that atomoxetine improves symptoms of ADHD compared to placebo after 12 weeks of treatment on the mean change from baseline to endpoint as measured by:

− Clinical Global Impression-ADHD-Severity (CGI-ADHD-S)

− CAARS Self Report (CAARS-S:SV)

− Patient Global Impression – Improvement (PGI-I)

• Montgomery Asberg Depression Rating Scale (MADRS): To assess the effect of atomoxetine in treating symptoms of depression in adults with ADHD as measured by the mean change in the total score after 12 weeks of treatment.

• Beck Anxiety Inventory (BAI): To assess the effect of atomoxetine in treating symptoms of anxiety in adults with ADHD as measured by the mean change in the total score after 12 weeks of treatment.

• To assess the correlation between the improvements on the AAQOL-29 Total Score with symptom reduction as measured by improvement on the CAARS- Inv:SV total ADHD symptom score.

***6.2.3. Secondary Functional Outcome Objectives***

• To test the effect of atomoxetine for the following measures compared to placebo:

− Habits Timeline Followback (TLFB) (Alcohol, Nicotine, Caffeine, Marijuana, and Drugs): the incidence of use for each of the following habits separately: alcohol, nicotine, caffeine, marijuana, and drugs as measured by the mean change

− Fagerstrom Test for Nicotine Dependence

− Social Adaptation Self-Evaluation Scale (SASS)

− Driving Behavior Survey Self-Report

− Driving Behavior Survey - Other Report

− Behavior Rating Inventory of Executive Function-Adult Version Self

Report (BRIEF-A)

− Epworth Sleepiness Scale

• To assess baseline smoking status as a predictor of response (a 25% decrease from baseline on the CAARS-Inv:SV total ADHD symptom score) and strong response (a 40% decrease from baseline on the CAARS-Inv:SV total ADHD symptom score ) to atomoxetine treatment compared with placebo.

**7. Investigational Plan**

**7.1. Summary of Study Design**

Study B4Z-US-LYDZ(b) is a multi-center, 12-week, randomized, placebo-controlled, double-blind, trial with 3 study periods for the evaluation of safety and efficacy between atomoxetine and placebo in approximately 440 adult patients (age 18 to 30) with ADHD at approximately 35 sites in the United States and Puerto Rico.

The study design employs a blinded, randomized placebo lead-in to blind patients and investigators to the start of active therapy (see discussion on the rationale for this design in Section 7.2). This protocol contains a description of the blinded study design with sufficient information to ensure patient safety. There will be no circumstances under which investigators or site personnel will know the complete details of the study design. A separate document (IRB Supplement 1) contains a complete account of the study design. IRB Supplement 1 will be provided along with this protocol to investigators’ ethical review boards and to regulatory agencies for review.

The protocol design contains a community sample (CS) which is a superset of subjects who do not qualify or qualify but choose not to participate in the randomized research clinical trial (RCT). Non-interventional health outcomes data will be collected from CS participant via web-based self-reporting done through the study website. The participants of the CS will interface only with the study website and will be independent of the RCT and investigator trial sites. The CS is further described in Protocol Attachment LYDZ(b).5.

**Pre-Screening Period**

**Study Period I Screening**

**Study Period II**

**12 weeks**

**Study Period III**

**12 weeks**

Outpatient Site

Screening

Passed screen, site available, & willing to participate in research clinical trial

**ALL SUBJECTS Web-based ADHD**

**& Study Screen**

**Atomoxetine 40-100mg** *(Dosed BID)*

**Placebo**

Randomization and transition to active study drug for ATX group will occur between Visit 2 and 4

**Patients completing SPII Atomoxetine OPEN-LABEL**

**36-100mg (***Dosed QD or BID)*

**Community Sample***

**Visit: 1 2 4 6 9**

*****Community Sample** includes only subjects who:

▪ Meet ADHD criteria and meet Incl/Excl screen, & willing to participate in research clinical trial, NO site available

▪ Meet ADHD criteria and Incl/Excl screen but opt out of research clinical trial

▪ Meet ADHD criteria but fails Incl/Excl Screen

ATX = Atomoxetine

**Figure LYDZ(b).1. Illustration of study design of B4Z-US-LYDZ(b)**

This study utilizes web-based screening assessments, web-based informed consent documents (electronic signature), and web-based patient-rated scales (e-PROs) for both the RCT and CS.

**Study Period I:** A screening period of 5 to 28 days to determine eligibility for the double-blind acute treatment phase.

Web Screening**:** Subjects interested in participating in the study will be evaluated through the following web-based screening process:

• The website will require the subject to opt-in (opt-in is a form of consent) prior to completing the first level of screening which is to determine the likelihood of meeting ADHD diagnostic criteria.

o Recruitment of subjects may occur through a variety of methods that include but are not limited to: centralized advertising which will direct subjects to the website, ADHD databases, investigator databases, or local (investigator level) advertising. Subjects recruited from investigator databases or site- level advertising will also be directed to the study website as the “first-step” of screening for the study.

• Subjects will complete an internet-based non-identifiable Adult ADHD Self- Report Scale (ASRS) to determine the likelihood of meeting ADHD diagnostic criteria. A raw score of 14 or greater (screen positive) is required for the subject to be eligible to proceed to the next level of screening.

• If the subject  **screens positive** for the web-based ASRS criteria for ADHD:

o The website will require the subject to opt-in prior to answering additional inclusion/exclusion criteria questions via the study website. After the subject opts-in, the subject is then presented with questions to answer regarding

other **study inclusion/exclusion criteria** (including the Patient Health Questionnaire) to determine if the subject meets eligibility to proceed with further evaluation for the RCT by a clinical trial site.

o Subjects who meet other inclusion/exclusion trial criteria AND for whom a site is available in their geography will be eligible to enter the RCT.

o Subjects deemed eligible for the RCT will be presented with site contact information for the investigator(s) participating in their region. Additionally, with subject permission, contact information for subjects eligible for the

RCT will be provided to sites within the geography of the eligible subject. That site will contact the eligible subject to schedule further evaluation (labs, etc) by the clinical trial site to determine if the subject fully meets all study inclusion/exclusion criteria.

• Subjects who  **screen positive** for the ASRS criteria for ADHD but  **do not qualify**

for the RCT for the following reasons:

o Do not meet full inclusion/exclusion criteria for the RCT (as determined by the web-based inclusion/exclusion questionnaire),

o Meet full inclusion/exclusion criteria per the web-based screening but do not have a site located within their geography, OR

o Meet full inclusion/exclusion criteria for the RCT per the web-based screening but do not wish to participate in the RCT,

***will be eligible to participate in the CS*** (described in Protocol Attachment LYDZ(b).5). Subjects participating in the CS interface only with the study website and are independent of the investigator clinical trial site.

RCT Site Screening**:** At or before Visit 1, informed consent for the RCT will be obtained by the investigator site before any study procedures are conducted and before patients discontinue any excluded medications. An informed consent document (ICD) approved by an ERB or similar body will be signed by the patient and/or representative deemed appropriate according to local laws and regulations. Information from assessment questionnaires and medical history will be recorded in the source documents and/or in the electronic data capture system. Patients will undergo laboratory tests, an ECG

evaluation, a psychiatric evaluation, and a physical examination with a patient history being recorded. Protocol Attachment LYDZ(b).1 contains a complete list of assessment procedures. Clinical assessments conducted during the screening period will include clinical history, Structured Clinical Interview for DSM-IV Axis Disorders-Research Version (SCID-RV; First et al, 2000), and the Adult ADHD Clinician Diagnostic Scale version 1.2 (ACDS v1.2). The CAARS-Inv:SV will also be administered and if the patient’s CAARS-Inv-SV total ADHD symptom score decreases more than 25% between Visit 1  **and** Visit 2, the patient will be discontinued. **Note:** The MADRS will also be administered and a *baseline* score of 3 or greater on Item 10 (Suicidal Thought) of the MADRS is an automatic exclusion from the trial. Exclusion item number [20] outlines details regarding Item 10 of the MADRS administered post-baseline.

If necessary for scheduling or other reasons, the tasks for Visit 1 can be accomplished on

2 separate clinic days. In this case, the date of the visit will be the first of the 2 clinic days and the 2 clinic days must not be separated by more than 14 days. However, if washout of an excluded medication is required, the clinic days can be separated by the time required for appropriate washout of the medication. If the time between Visit 1 and Visit 2 exceeds the visit window interval to allow for washout of medications, this will not be considered a protocol violation.

**Washout.** For patients who have been taking medications excluded by this protocol, the following guidelines for medication washout will apply.

(Special note: informed consent must be obtained prior to beginning any medication washout-even if the washout is commenced prior to Visit 1).

• If the patient is on medication requiring washout on the first day of Visit 1, the ECG and laboratory examinations should be obtained on the second clinic day of Visit 1. The patient must be medication-free for at least 5 half-lives of the drug. The first clinic day will be considered the Visit 1 date.

• Patients who enter the study at Visit 1 taking stimulant medications for the treatment of ADHD must be medication-free for at least 24 hours immediately prior to Visit 2 before diagnostic baseline data/measures are obtained. These medications will also need to be discontinued for the duration of the trial.

• Patients who are taking any medication excluded by the protocol at Visit 1 that has a half-life of >24 hours must have a washout equal to or greater than 5 half- lives of the parent compound and any active metabolite of the parent compound immediately prior to Visit 2.

• Patients who are taking SSRIs at Visit 1 must have a washout equal to or greater than 5 half-lives of the parent compound and any active metabolite of the parent compound  **or** for 2 weeks, whichever is  **longer**, immediately prior to Visit 2.

• Patients who are taking any psychotropic medications that have a parent and/or active metabolite half-life requiring > 28 days of washout must have their medications reviewed by the Eli Lilly and Company research physician who is responsible for monitoring the study.

• Patients who are taking any health food supplements that, in the investigator’s opinion, may have central nervous system activity (for example, St. John’s Wort, melatonin, etc.) must have a washout equal to a minimum of 5 half-lives of that supplement immediately prior to Visit 2. If the half-life of the supplement is unknown, then the patient should have a 28-day washout.

At Visit 2, patients will be further evaluated for inclusion/exclusion criteria and, patients meeting requirements will be “enrolled” in the study and receive study therapy. Patients who do not meet all entry criteria at Visit 2 will be discontinued from the study.

**Note**: *patients who do not meet criteria for the RCT after screening at an investigative site (screen failure) are* ***not*** *eligible for the CS described in Protocol Attachment LYDZ(b).5.*

**Study Period II:** A 12-week acute phase of double-blind treatment. Patients will be assessed 2 weeks after Visit 2 and then every 3 weeks until Visit 5 then at 4 weeks (Visit

6, the end of acute phase of study). Patients who meet all screening criteria will be

dispensed blinded medication and instructed to begin doses the morning following Visit

2. Patients will be randomized 1:1 to either atomoxetine or placebo some time between

Visit 2 and 4. Since the time at which randomization occurs is double-blinded, neither

the investigator nor the patient will know when randomization occurs. Further, because of the potential for a double-blind placebo lead-in, any patient randomly assigned to atomoxetine may receive placebo some time during the potential lead-in phase. Patients in the atomoxetine group may receive atomoxetine as early as Visit 2 or as late as Visit 4. The study drug is to be taken by the patient twice-daily, once in the morning and once in the afternoon or early evening. It is strongly recommended to take the study medication with food. Patients randomized to atomoxetine will begin treatment with 40 mg/day (dosed 20 mg twice daily [BID]) for a minimum of 7 days. Immediately following the last dose of 40 mg/day (dosed 20 mg BID), the patient will receive 80 mg/day (dosed 40 mg BID) atomoxetine for a minimum of 7 days. Following Visit 3, one unscheduled

dose decrease is allowed if needed for tolerability or safety. Only one dose decrease will

be permitted during Study Period II.

Starting at Visit 5, if in the judgment of the investigator, the patient has significant residual symptoms, the dose can be increased to the maximum dose of 100 mg/day (dosed 50 mg BID). However, patients who previously required a dose decrease due to safety or tolerability will not be permitted to have a dose increase.

The unscheduled dose decrease or dose increase will only be allowed by one dose level (that is, 80 mg/day to 40 mg/day, 100 mg/day to 80 mg/day, or 80 mg/day to 100 mg/day). Patients unable to tolerate 40 mg/day (dosed 20 mg BID) should be discontinued. Efficacy data and health outcome measures as outlined in the schedule of events will be evaluated.

If the patient has access to the internet, the patient-rated scales (electronic patient- reported outcomes [e-PROS]) may be completed by the patient outside of the investigator’s office. The patient will be permitted to log into the study website 3 days prior to the originally scheduled visit to begin completing patient-rated questionnaires. The patient may log in and out of the study website without losing any data captured at

the previous login session. If the patient has not completed all of the patient-rated scales on the day of the originally scheduled clinic visit, the patient must complete them at the investigator’s site by the end of the clinic visit. If the patient reschedules the clinic visit, the rescheduled date must occur within the visit interval described in Protocol Attachment LYDZ(b).1. The patient will still be permitted to complete the e-PROs prior to the rescheduled clinic visit IF the visit is within the visit interval window.

**Study Period III:** A 12-week open-label extension period. Patients completing Study Period II will be eligible to enter Study Period III. Patients will be administered open- label atomoxetine at doses of 36 to 100 mg/day that can be taken as QD or BID dosing starting the day after Visit 6.

**7.2. Discussion of Design and Control**

Study Period I is designed to determine if subjects meet all of the inclusion criteria and none of the exclusion criteria. The screening interval may last between 5 to 28 days in order to accommodate the long washout period of some excluded medications, such as fluoxetine. Where no washout is required, qualified patients may enroll 5 days after they have completed all screening and laboratory assessments.

Study Period II is designed to assess the safety and efficacy of atomoxetine compared to placebo on signs and symptoms of ADHD in young adults over 12 weeks of therapy.

This study design uses a blinded randomization placebo lead-in period which blinds the patients and investigational sites to the start of active therapy. The rationale for blinding the start of active therapy is based on the experience of researchers at Lilly Research Laboratories and on the published literature which underscores the difficulties of detecting a true drug effect in the face of the typically sizable placebo response.

Consequently, efforts to satisfactorily maintain the double-blind nature of the study are of the utmost importance. Evidence suggests that using a 1-week, single-blind, placebo

lead-in period, a standard approach for antidepressant clinical trials, is of limited value in eliminating subjects most susceptible to volatile symptom rating and high placebo response rates (Trivedi et al. 1994).

This study design method blinds investigators and patients to the time of initiation of the active therapy. Blinding the time of initiation of the active therapy will ensure a more accurate baseline assessment, and therefore, will obtain more accurate evaluation for the treatment effect. The blinding of start of active study drug, as well as assignment of patients to treatment groups, will be maintained by use of an interactive voice response system (IVRS).

This protocol contains a description of the study design in sufficient detail to inform the investigators and staff personnel of the study goals and to ensure patient safety. Under no circumstances will investigators or staff personnel know the complete details of the study design. A separate document (IRB Supplement 1) contains a complete account of the study design. IRB Supplement 1 will be provided along with this protocol to investigators’ ethical review boards and regulatory agencies for review.

The starting dose of atomoxetine 40 mg/day (dosed BID) for 7 days during Study Period II was selected in order to improve tolerability for some patients who experience expected pharmacologic effects such as gastrointestinal upset. Further, this dosing strategy is within label and most closely mirrors clinical practice.

Study Period III is a 12-week open-label extension period available for patients completing SPII.

**8. Study Population**

After the completion of screening during Visit 1 and Visit 2, patients who are not excluded and continue to meet all of the inclusion criteria will be eligible to begin the double-blinded treatment period at the end of Visit 2.

Study participants should be instructed not to donate blood or blood products during the study and for 1 week following the study.

**8.1. Inclusion Criteria**

Patients are eligible to be included in the study only if they meet **all** of the following criteria:

[1] Currently meets DSM-IV™-TR criteria for ADHD as assessed by the ACDS.

Concomitant Axis I diagnoses that are also allowed (current - if diagnosed greater than 6 months immediately prior to Visit 1 - or lifetime) are specific phobias, Generalized Anxiety Disorder (GAD), and Social Anxiety

Disorder. Current or lifetime dysthymia is allowed if diagnosed greater than

2 years immediately prior to Visit 1.

[2] Patient’s CAARS-Inv:SV total ADHD symptom score cannot decrease by more than 25% between Visit 1  **and** Visit 2.

[3] Patients must have a CGI- ADHD-S score of 4 (moderate symptoms) or greater at both Visit 1  **and** Visit 2.

[4] Male or female outpatients 18 to 30 years of age at Visit 1.

[5] (Note: This inclusion criterion applies only to females of childbearing potential—those women not surgically sterilized and between menarche and

1 year post-menopause). Women of childbearing potential must test negative for pregnancy at the time of enrollment based on a serum pregnancy test and agree to use a reliable method of birth control (for example, use of oral contraceptives or Norplant®; a reliable barrier method of birth control: diaphragms with contraceptive jelly, cervical caps with contraceptive jelly, condoms with contraceptive foam; intrauterine devices; partner with vasectomy; or abstinence) during the study.

[6] Must have an ECG performed at Visit 1. The results of the screening ECG must be read and the results must be available to the investigator or qualified designee at Visit 1. If an ECG shows an abnormality meeting the criteria in the protocol, the guidelines in the attachment for excluding, discontinuing

the patient and contacting the sponsor should be followed. Patients with other abnormalities may be included at the discretion of the investigator; however, the Lilly physician monitor or designee must be notified.

[7] Must be able to swallow capsules.

[8] No evidence of cognitive impairment that would preclude adequate participation in the trial or ability to sign the informed consent document.

[9] Have been judged by the investigator to be reliable to keep appointments for clinic visits and all tests, including venipuncture, and examinations required by the protocol.

[10] Must be able to communicate effectively with the investigator and study coordinator in English; specifically, patients must possess an educational level and degree of fluency of English that enables them to independently complete study tasks (for example, read and understand informed consent, complete self-rated study scales, etc.), and communicate suitably with the investigator and study coordinator as judged by the investigator. Further, patients must be willing to complete some questionnaires on a computer via the study website.

***8.1.1. Disease Diagnostic Criteria***

Pre-screening assessment conducted prior to a patient presenting at a clinical research site will include the ASRS v1.1. Clinical assessments conducted during the screening period will include clinical history, SCID-RV (First et al. 2000), and the ACDS v1.2.

**8.2. Exclusion Criteria**

Patients will be excluded from the study if they meet **any** of the following criteria:

[11] Are investigator site personnel directly affiliated with this study and/or their immediate families. Immediate family is defined as a spouse, parent, child, or sibling, whether biological or legally adopted.

[12] Are Lilly employees.

[13] Are currently enrolled in, or discontinued within the 30 days prior to Visit 1 from, a clinical trial involving an off-label use of an investigational drug or device (other than the study drug used in this study), or concurrently

enrolled in any other type of medical research judged not to be scientifically

or medically compatible with this study.

[14] Patients who have not responded to an adequate trial of atomoxetine, or have previously been adequately treated with atomoxetine, who have experienced intolerable side effects while receiving atomoxetine, OR have previously completed or withdrawn from this study or any other study investigating atomoxetine.

[15] Initiation of psychotherapy, change in intensity of psychotherapy, cognitive behavioral therapy, supportive therapy, or other non-drug therapies (such as acupuncture or hypnosis) within 30 days immediately prior to Visit 1, or at any time during the study.

[16] Any of the following current DSM-IV-TR™ Axis I diagnoses including: patients diagnosed with major depressive disorder (MDD), panic disorder, post-traumatic stress disorder, or an eating disorder within the past year immediately prior to Visit 1.

Any of the following current or lifetime DSM-IV-TR™ Axis I diagnoses including: patients diagnosed with obsessive-compulsive disorder, bipolar affective disorder, or psychosis.

[17] Are pregnant or are breastfeeding.

[18] Have organic brain disease, for example, dementia, or traumatic brain injury residua. Have a history of any seizure disorder (other than febrile seizures) or patients who have taken (or are currently taking) anticonvulsants for seizure control.

[19] Have a history of severe allergies to more than 1 class of medications or multiple adverse drug reactions.

[20] Patients judged clinically to be at serious suicidal risk, or patients, in the opinion of the investigator, are poor medical or psychiatric risks for study completion.

Specifically, a **baseline score of 3 or greater** on Item 10 (Suicidal Thought)

of the MADRS scale **is an automatic exclusion** from the trial.

A **post-baseline** spontaneous report of suicidal thoughts or behavior at any visit OR a score of **2 or greater** on Item 10 (Suicidal Thought) of the MADRS scale at Visit 4, 6, and 9 will require the administration of the Columbia Suicide-Severity Rating Scale.

If in the PI’s clinical judgment, the PI determines the patient to *not* be at serious suicide risk (using Columbia scale as a tool), the patient may continue in the study.

If in the PI clinical judgment, the PI determines the patient to be at serious suicidal risk, the risk should be addressed immediately using the PI’s best clinical judgment. Further,

a. serious suicidal risk is exclusionary and the patient must be discontinued, and

b. the site must contact and inform the sponsor of the event within 24 hours by contacting the Clinical Research Associate (CRA) who will then contact the Lilly study physician.

[21] Are currently using alcohol, drugs of abuse, or any prescribed or over-the- counter medication in a manner which the investigator considers indicative of DSM-IV-TR™ criteria for substance abuse or dependence.

If the urine drug screen is positive for any substance, the investigator should discontinue the patient *only* if the substance is being used in a manner indicative of DSM-IV-TR™ criteria for substance abuse or dependence OR

if the substance is on the excluded medication list (Protocol Attachment

LYDZ(b).3) and the patient cannot or will not discontinue the medication.

[22] Are taking any psychotropic medication on a regular basis, including health- food supplements that the investigator feels have central nervous system activity (for example, St. John’s Wort, melatonin, etc.).

[23] Have a current diagnosis of uncontrolled hyper- or hypothyroidism. Patients with an initial abnormal TSH screen should be evaluated with additional

thyroid tests through a local lab to determine eligibility for the study or the need for treatment prior to re-screening and participation in the study. **Note:** Patients previously diagnosed with hyperthyroidism or hypothyroidism that have been treated on a stable dose of thyroid supplement for at least the past

3 months immediately prior to Visit 1, have medically appropriate TSH

concentrations, and are clinically euthyroid are allowed.

[24] Have any medical condition that would increase sympathetic nervous system activity markedly (for example, catecholamine-secreting neural tumor), or who are taking a medication on a daily basis (for example, albuterol, inhalation aerosols, pseudoephedrine), that has sympathomimetic activity. Such medications can be taken on an as-needed basis.

[25] Have used monoamine oxidase inhibitors (MAOIs) during the 2 weeks

(14 days) prior to Visit 2.

[26] Have a current diagnosis of uncontrolled hypertension. For the purposes of this protocol, hypertension will be defined as average systolic or diastolic blood pressure, measured on at least 2 separate occasions, greater than or equal to 140/90. Patients whose blood pressure is controlled with non- excluded medication are eligible for inclusion.

[27] Who have narrow-angle glaucoma.

[28] Have a history of difficulty starting a stream of urine or other symptoms suggestive of prostate enlargement, or other evidence of urinary hesitancy on clinical history.

[29] Who anticipate a move outside the geographic range of the investigative site after beginning active treatment, or who plan extended travel inconsistent with the recommended visit interval during Study Period II.

[30] Serious medical illness, including any cardiovascular, hepatic, renal, respiratory, hematologic, endocrinologic, or neurologic disease, or clinically significant laboratory abnormality that is not stabilized or is anticipated to require hospitalization within 6 months, in the opinion of the clinical investigator. Clinically significant laboratory abnormalities are those that, in the judgment of the investigator, indicate a serious medical problem.

[31] Have had transcranial magnetic stimulation (TMS) or history of psychosurgery, electroconvulsive therapy (ECT), or vagus nerve stimulation within 12 months immediately prior to Visit 1.

[32] Are unsuitable in any way to participate in this study, in the opinion of the investigator.

***8.2.1. Rationale for Exclusion of Certain Study Candidates*** Exclusion Criteria [11] and [12] reduce the potential bias that may be introduced at the study site. Exclusion Criterion [13] eliminates drugs that cannot be mapped to a standard drug dictionary, or for which little data are known to analyze the potential relationship of adverse events or drug interactions. Exclusion criteria [14-17], [21-23], and [29] eliminate conditions, situations, or medications that may interfere with protocol compliance or confound the study data. All other exclusion criteria [18-20], [24-28], and [30-32] are present to ensure the safety of study patients

No patients are excluded from participation based on race, ethnicity, or gender. Patients

at serious suicidal risk are excluded to ensure patient welfare. Exclusions of patients with specific medical conditions and/or patients taking specific medications are listed because of potential for interaction of those conditions or medication with known or potential effects of atomoxetine. Patients taking certain psychoactive medications or drugs of

abuse noted in the exclusion criteria are excluded because of the potential for these

medications to confound interpretation of the efficacy results.

**8.3. Discontinuations**

***8.3.1. Discontinuation of Patients***

The criteria for enrollment must be followed explicitly. If a patient who does not meet enrollment criteria is inadvertently enrolled, that patient should be discontinued from the study, and Lilly or its designee must be contacted.

In addition, patients will be discontinued from the study drug and from the study in the following circumstances:

• Enrollment in any other clinical trial involving an off-label use of an investigational drug or device or enrollment in any other type of medical research judged not to be scientifically or medically compatible with this study.

• If Exclusion Criterion [11-32] is observed, or develops, after entry or enrollment. In this case, the patient will be discontinued from the study at the next visit or sooner in the event of a safety exclusion criterion.

• The investigator decides that the patient should be withdrawn. If this decision is made because of a serious adverse event or a clinically significant laboratory value, the study drug is to be discontinued and appropriate measures are to be taken. Lilly or its designee is to be alerted immediately. Refer to Section 10.3, Safety Evaluations.

• The patient or attending physician requests that the patient be withdrawn from the study.

• The patient, for any reason, requires treatment with another therapeutic agent that has been demonstrated to be effective for treatment of the study indication. In this case, discontinuation from the study occurs prior to

introduction of the new agent.

• The investigator or Lilly, for any reason, stops the study or stops the patient's participation in the study.

Patients who discontinue the study early will have end-of-study procedures performed as shown in the Study Schedule (Protocol Attachment LYDZ(b).1).

***8.3.2. Discontinuation of Study Sites***

Study site participation may be discontinued if Lilly, the investigator, or the ethical review board of the study site judges it necessary for any reason.

***8.3.3. Discontinuation of the Study***

The study will be discontinued if Lilly judges it necessary for any reason.

**9. Treatment**

**9.1. Treatments Administered**

***9.1.1. Study Period II***

This study involves a comparison of atomoxetine 40-100 mg/day administered orally, twice daily (BID) with placebo. Patients who meet all screening criteria will be dispensed blinded medication and instructed to begin doses the morning following Visit

2. Patients will be randomized 1:1 to either atomoxetine or placebo some time between Visit 2 and 4. Since the time at which randomization occurs is double-blinded, neither the investigator nor the patient will know when randomization occurs. Further, because of the potential for a double-blind placebo lead-in, any patients randomly assigned to atomoxetine may receive placebo some time during the potential lead-in phase. Thus,

patients in the atomoxetine group may receive atomoxetine as early as Visit 2 or as late as

Visit 4. The study drug is to be taken by the patient twice-daily, once in the morning and once in the afternoon or early evening. It is strongly recommended to take the study medication with food.

Patients randomized to atomoxetine will:

• begin treatment with atomoxetine 40 mg/day dosed BID for a minimum of 7 days; then,

• the patient will receive atomoxetine 80 mg/day dosed BID for a minimum of 7 days.

Following Visit 3, one unscheduled dose decrease is allowed if needed for tolerability or safety. Only one dose decrease will be permitted during Study Period II.

Starting at Visit 5, if in the judgment of the investigator, the patient has significant residual symptoms, the dose can be increased to the maximum dose of 100 mg/day (dosed 50 mg BID). However, patients who previously required a dose decrease due to safety or tolerability will not be permitted to have a dose increase.

The unscheduled dose decrease or dose increase will only be allowed by one dose level (that is, 80 mg/day to 40 mg/day, 100 mg/day to 80 mg/day, or 80 mg/day to 100 mg/day). Patients unable to tolerate 40 mg/day (dosed 20 mg BID) should be discontinued.

The investigator or his/her designee is responsible for explaining the correct use of the investigational agent(s) to the patient, verifying that instructions are followed properly, maintaining accurate records of study drug dispensing and collection, and returning all unused medication to Lilly or its designee at the end of the study.

Patients will be instructed to contact the investigator as soon as possible if he or she has a complaint or problem with the study drug so that the situation can be assessed.

***9.1.2. Study Period III***

All eligible patients who complete the double-blind Study Period II will have the option

of participating in a 12-week open-label extension period. In Study Period III, study drug

(atomoxetine) will be dispensed to all patients.

Dosing of atomoxetine during the open-label period will commence the morning immediately following the last dose of blinded medication. Dosing may be once daily or twice-daily administration. It is strongly recommended to take the study medication with food.

The starting dose of atomoxetine will be initiated at 7 days of 40 mg/day followed by 7 days of 80 mg/day for those dosed once daily. For those patients prescribed BID dosing in the open-label period, 18 mg capsules were chosen for the lowest starting dose as 20 mg capsules are not available in the marketed form of atomoxetine.

After the initial titration period, the dose may be titrated freely (that is, the dose may be increased or decreased at any time) between 36 mg/day and 100 mg/day during the remainder of Study Period III depending on the patient’s clinical response.

**9.2. Materials and Supplies**

**Table LYDZ(b).1. Clinical Study Materials Informationa**

**Test Material Source Dose Strength Packaging**

**Study Period II** Atomoxetine hydrochloride

Eli Lilly and Co. Indianapolis, IN

20 mg

25 mg

40 mg

Capsules in blister cards, blister packs,

or bottles

Placebo Eli Lilly and Co.

Indianapolis, IN

N/A Capsules in blister cards, blister packs,

or bottles

**Study Period III**

Atomoxetine hydrochloride

Eli Lilly and Co. Indianapolis, IN

18 mg

40 mg

60 mg

Market Image

Bottles

**a** All study materials should be stored at room temperature.

Study drug will be labeled in accordance with Lilly Standard Operating Procedures and the applicable guidelines and regulations of the United States of America where the study is to be conducted. Proper measures will be taken to ensure atomoxetine and placebo capsules are indistinguishable.

The investigator or his/her designee is responsible for explaining the correct use of the investigational agent(s) to the patient, verifying that instructions are followed properly, maintaining accurate records of study drug dispensing and collection, and returning all unused medication to Lilly or its designee at the end of the study.

Patients will be instructed to contact the investigator as soon as possible if he or she has a complaint or problem with the study drug so that the situation can be assessed.

**9.3. Method of Assignment to Treatment**

Patients who meet all criteria for enrollment will be randomized 1:1 to double-blind treatment sometime between Visit 2 and Visit 4. However, because of the potential for a double-blind placebo lead-in and because any patients randomly assigned to atomoxetine may receive placebo some time during the potential lead-in phase, patients in the atomoxetine group may receive atomoxetine as early as Visit 2 or as late as Visit 4. During the double-blind treatment period of the study (Study Period II) assignment to treatment groups will be determined by a computer-generated random sequence using an interactive voice response system (IVRS). The IVRS will be used to assign packages containing double-blind study drug to each patient.

During the open-label extension period of the study (Study Period III) market-image atomoxetine will be supplied by Lilly.

To achieve between group comparability for site factors, patients will be randomized at the site level.

**9.4. Rationale for Selection of Doses in the Study**

The atomoxetine dose of 40 mg/day to 100 mg/day in the double-blinded period of the study is based on previous data from ADHD trials. Results of clinical studies of atomoxetine in adults with ADHD suggest that most patients require doses >60 mg/day, and that many adults require doses up to 100 mg/day. Doses of atomoxetine up to 100 mg/day have been studied as both single and divided doses without evidence of any serious safety concerns. The maximum dose to be administered in this study is 100 mg/day for any patient regardless of weight.

For patients prescribed BID dosing in the open-label extension period, 18 mg capsules were chosen for the lowest starting dose as 20 mg capsules are not available in the marketed form of atomoxetine.

**9.5. Selection and Timing of Doses**

The dose of atomoxetine was selected based on experience in several clinical trials in children, adolescents, and adults. Atomoxetine has been shown to be efficacious for ADHD at doses up to 100 mg/day, and has been efficacious whether the total daily dose

is given once daily or divided into two separate doses. The regimen of twice-daily dosing

was chosen because data from B4Z-US-LYBM (a study in adults with ADHD that compared BID dosing to QD dosing) showed a small, but significant advantage in treating the symptoms of ADHD when dosing was administered BID (B4Z-US-LYBM, data on file, Eli Lilly & Company). The atomoxetine doses in this study are consistent with the FDA label recommendations for the treatment of ADHD.

***9.5.1. Special Treatment Considerations***

Atomoxetine is to be taken twice-daily during the double-blind phase (Study Period II). The first dose should be taken in the morning and the second dose should be taken in the afternoon or early evening. Atomoxetine may be taken once daily or twice daily during the extension phase (Study Period III). Atomoxetine should be swallowed whole; the capsules should not be crushed, broken or opened. Atomoxetine is satisfactorily absorbed regardless of whether it is taken with or without food. It has been shown to be equally effective if the patient has eaten prior to taking the medication or not. However, some data suggest that tolerability is better when atomoxetine is administered with food. Therefore, it is strongly recommended to take the study medication with food.

**9.6. Blinding**

Study Period II is the double-blind phase of the study.

To preserve the blinding of the study, a minimum number of Lilly personnel will see the randomization table and treatment assignments before the study is complete.

Emergency unblinding for adverse events may be performed through an IVRS, which may supplement or take the place of emergency codes generated by a computer drug- labeling system. This option may be used ONLY if the patient's well-being requires knowledge of the patient’s treatment assignment. All calls resulting in an unblinding event are recorded and reported by the IVRS. All codes, whether sealed or opened, must be returned to Lilly upon completion of the study.

The investigator should make every effort to contact the Lilly clinical research physician prior to unblinding a patient's treatment assignment. If a patient's treatment assignment is unblinded, Lilly must be notified immediately by telephone.

If an investigator, site personnel performing assessments, or patient is unblinded, the patient must be discontinued from the study. In cases where there are ethical reasons to have the patient remain in the study, the investigator must obtain specific approval from a Lilly clinical research physician for the patient to continue in the study.

**9.7. Concomitant Therapy**

In general, concomitant medications with primarily central nervous system (CNS)

activity are not allowed in this protocol.

Protocol Attachment LYDZ(b).3 lists commonly used drugs, which may or may not be used acutely or for longer periods during Study Period II and Study Period III. All concomitant medications will be recorded on electronic case report forms (e-CRFs), including dose and duration of treatment.

Any changes to this list will be communicated to investigators and will not constitute a protocol amendment. **Patients must sign the informed consent document before stopping any excluded medications, if the reason they are stopping the excluded medications is for the sole purpose of being allowed to enter the study.**

Patients requiring excluded concomitant drugs will be discontinued from the study, unless approval has been obtained from the Lilly physician monitor or Lilly’s designated representative.

Patients are not allowed to take medications for sedation or sleep during the study.

**9.8. Treatment Compliance**

Patient compliance with study medication will be assessed at each visit. Compliance will be assessed by direct questioning and counting returned capsules.

Patients who are significantly noncompliant will be discontinued from the study. A patient will be considered *compliant* during a visit interval if he or she took the prescribed medication at the prescribed dose on at least 75% of the days in the visit interval. Similarly, a patient will be considered significantly *noncompliant* if he or she intentionally or repeatedly takes less than the prescribed amount (75%) of medication or more than the prescribed amount (120%) as judged by the investigator.

If a patient is not compliant during a visit interval, the patient will be counseled regarding the importance of compliance in this study. The patient may be discontinued at this time if, in the opinion of the investigator, the patient is deemed unlikely to become compliant.

The second time that a patient is noncompliant with his or her dosage schedule; he or she must be discontinued from the study unless noncompliance results from unforeseen circumstances (that is, blizzard, illness). In such instances, the Lilly research physician who is responsible for monitoring the study may grant permission for the patient to remain in the study.

**10. Efficacy, Health Outcome/Quality of Life Measures, and Safety Evaluations, and Appropriateness of**

**Measurements**

Study procedures and their timing (including tolerance limits for timing) are summarized in the Study Schedule (Protocol Attachment LYDZ(b).1).

**10.1. Efficacy Measures**

***10.1.1. Primary Efficacy Measure***

**10.1.1.1. Conners’ Adult ADHD Rating Scale-Investigator Rated: Screening**

**Version (CAARS-Inv:SV)**

The primary efficacy measure for this study is the CAARS-Inv: SV. This scale will be administered at every visit.

The CAARS-Inv: SV is a 30-item scale containing 3 subscales: the Inattention subscale

(items 1, 9, 13, 14, 19, 21, 26, 29, and 30), the Hyperactivity-Impulsivity subscale (items

2, 4, 6, 8, 16, 18, 22, 25, and 27), and the ADHD Index (items 3, 5, 7, 10, 11, 12, 15, 17,

20, 23, 24, and 28) (Conners et al. 1999). The 18-item total ADHD symptom score is the sum of the Inattention and Hyperactivity-Impulsivity subscales. Each individual item is scored on a 0 to 3 scale (0 = not at all, never; 1 = just a little, once in a while; 2 = Pretty much, often; 3 = very much, very frequently). The rating scale will assess the symptom severity over the past week. The scale will be administered and scored by qualified personnel at the investigative site. The Inattention subscale, Hyperactivity/Impulsivity subscale, ADHD Index subscale, and the 18-item total ADHD symptom score will be computed. The scale is scored by a qualified rater based on an interview with the patient.

***10.1.2. Secondary Efficacy Measures***

The following secondary efficacy measures will be collected at the times shown in

Attachment LYDZ(b).1 (Study Schedule).

**10.1.2.1. Adult ADHD Quality of Life (AAQOL-29)**

The AAQOL-29 is a patient-reported outcome measure used to examine the disease- specific functional impairments and quality of life for adults with ADHD. The domains included in the AAQOL are life productivity, psychological health, quality of relationships, and life outlook. Consistent with the majority of existing quality of life measures, higher scores on the AAQOL-29 indicate better functioning. A longer version of this scale, which includes 80 items, was used in two atomoxetine studies: B4Z-MC- LYBV and B4Z-US-LYCU (Brod et al. 2005).

**10.1.2.2. Clinical Global Impression—ADHD-Severity (CGI-ADHD-S)**

The CGI-ADHD-S is a single-item clinician rating of the clinician’s assessment of the patient’s severity of the ADHD symptoms in relation to the clinician’s total experience with ADHD patients (Guy 1976; NIMH 1976).Severity is rated on a 7-point scale

(1 = normal, not at all ill; 7 = among the most extremely ill patients).

**10.1.2.3. Conners’ Adult Attention-Deficit/Hyperactivity Disorder Rating**

**Scale–Self Report**−**Screening Version-Administered (CAARS-S:SV*)*** The CAARS-S:SV is the same scale as the CAARS-Inv: SV, but it is completed by the patient. The individual items, subscales, and scoring will be the same as described for the CAARS-Inv: SV.

**10.1.2.4. Patient Global Impression-Improvement (PGI-I)**

The PGI-I is a 7-point scale modeled after the CGI on which patients rate any change in their overall status that they had experienced since beginning the study drug. The score on this scale ranges from 1 (very much improved) to 7 (very much worse).

**10.1.2.5. Montgomery Asberg Depression Rating Scale (MADRS)**

The MADRS (Montgomery et al. 1979) is a widely used rating scale for severity of depressive mood symptoms and is administered by the investigator. The scale consists of

10 items, each rated on a scale from 0 to 6. The MADRS total score is the sum of the 10 items and the score ranges from 0 to 60. Higher scores denote more severe depressive symptoms.

Item 10 (Suicidal Thoughts) is specifically evaluated to determine suicidal risk. For this study, a **baseline score of 3 or greater** on Item 10 (Suicidal Thought) of the MADRS scale is an automatic exclusion from the trial.

A **post-baseline** score of **2 or greater** on Item 10 (Suicidal Thought) of the MADRS scale at Visit 4, 6, and 9 will require the administration of the Columbia Suicide-Severity Rating Scale.

***10.1.2.5.1. Columbia Suicide-Severity Rating Scale:***

The Columbia Suicide-Severity Rating Scale is a physician administered scale to evaluate suicidal intent and plan and is used in this study as a *required tool* to evaluate serious suicidal risk. The scale evaluates 3 categories: suicidal behavior, suicidal ideation, and intensity of ideation. For the purposes of this study, it will be required as part of an algorithm should a patient score 2 or greater on Item 10 of the MADRS scale at Visit 4,

6, or 9 OR should a patient spontaneously report suicidal thoughts, plans, or behavior. This tool will assist the investigator to determine if the patient is at serious suicidal risk.

**10.1.2.6. Beck Anxiety Inventory (BAI)**

The BAI is a 21-item self-reported screening tool for measuring anxiety severity. Each item is rated on a 4-point Likert scale ranging from 0 (not at all) to 3 (severely; I could barely stand it). Each item is descriptive of subjective, somatic, or panic-related symptoms of anxiety. Patients record how much they have been bothered by each symptom during the past week, including the day the questionnaire is administered. The total score ranges from 0 to 63.

**10.2. Secondary Functional Outcome Measures**

***10.2.2. Habits Timeline Followback (TLFB): Alcohol, Nicotine, Caffeine, Marijuana, and Drugs***

The Habits Timeline Followback is a variation of the Alcohol Timeline Followback

(TLFB) which is a method for assessing the quantity of alcohol consumption on a daily basis (Sobell et al. 1992). In addition to assessing alcohol, the Timeline Followback will assess nicotine, caffeine, marijuana, and drug habits utilizing the same method. With a calendar as a guide, the interviewee provides a retrospective estimate of daily habits over

a specified period of as long as the previous year. The goal is to provide a detailed record of patterns of use that can be used to guide treatment and to assess treatment outcome (Sobell et al. 1996).

***10.2.3. Fagerstrom Test for Nicotine Dependence (FTND)***

The FTND was designed to provide an ordinal measure of nicotine dependence related to cigarette smoking (Fagerstrom and Schneider 1989). It contains items that evaluate the quantity of cigarette consumption, the compulsion to use, and dependence. The FTND contains 4 yes-no and 2 multiple choice questions and can be used in a self-report format. The items on FTND are scored 0 to 3 for multiple choice items, the items are summed to yield a total score of 0-10.

***10.2.4. Social Adaptation Self-Evaluation Scale (SASS)***

The SASS is a patient completed scale that consists of 21 items that examine behavior and subjective perception, including satisfaction, self-perception and motivation in participating in and maintaining relationships with family and friends, satisfaction in

work, home and leisure activities, and intellectual interests. Each item is scored from 0 to

3, corresponding to minimal and maximal social adjustment, with a total score range from 0 to 60 (Bosc et al. 1997).

***10.2.5. Driving Behavior Survey (Self-Report)***

The Driving Behavior Survey (Self-Report) is a 26 item self-rated driving survey. Examples of driving behaviors included in the survey are “putting on seat belt, driving within speed limits, yielding the right of way to other drivers.” Items are rated on a 4-

point scale (1 = not at all or rarely, 2 = sometimes, 3 = often, 4 = very often). The total score is the sum of the 26 items. A driving history is completed by self-report the first time a rater completes the Driving Behavior Survey (Self-Report). Examples of history items include “how many times have you: received a speeding ticket, been cited for reckless driving, and received a parking ticket.”

***10.2.6. Driving Behavior Survey (Other-Report)***

The Driving Behavior Survey (Other-Report) is a 26-item driving survey completed by someone other than the patient/driver. Examples of driving behaviors included in the survey match those listed in the Self-Report version of the scale. Items are rated on a 4- point scale (1 = not at all or rarely, 2 = sometimes, 3 = often, 4 = very often). The total score is the sum of the 26 items. If an observer is not available to complete this survey – the patient will not be disqualified or discontinued from the study. Similarly, if an

observer is identified but does not complete the Visit 2 rating scale, the patient may

identify a new observer. Note: If an observer rating is not collected at Visit 2, then the observer rating scale should not be collected at Visit 4 or Visit 6. The same observer

must complete the rating scales for Visits 2, 4, and 6. If the patient is unable or unwilling to identify a new observer, the patient will not be disqualified or discontinued from the study.

***10.2.7. Behavior Rating Inventory of Executive Function-Adult***

***Version (BRIEF-A)***

The BRIEF-A is a standardized measure that assesses adult executive functioning/self- regulation in his or her everyday environment. The self-report form is designed to be completed by adults 18-90 years of age, including adults with a wide variety of developmental, systemic, neurological, and psychiatric disorders such as attention disorders, learning disabilities, autism spectrum disorders, traumatic brain injury, multiple sclerosis, depression, mild cognitive impairment, dementia, and schizophrenia. The BRIEF-A consists of 75 items within nine non-overlapping theoretically and empirically derived clinical scales that measure various aspects of executive functioning (Mahone et al. 2002).

***10.2.8. Epworth Sleepiness Scale (ESS)***

The Epworth Sleepiness Scale is used to determine the level of daytime sleepiness (Johns

1991). The ESS is a self-rated questionnaire with 8 items that describe normative daily situations known to vary in their soporific qualities. Subjects rate the likelihood of dozing off or falling sleep in each of these situations. Each item is rated on a 4-point scale from 0 (would never doze) to 3 (high chance of dozing). The item scores are summed to produce a total score (range of 0-24). Score >10 (95th percentile) are considered to be suggestive of significant daytime sleepiness. Score >15 have been

associated with pathological sleepiness that may be due to specific conditions such as obstructive sleep apnea or narcolepsy.

**10.3. Safety Evaluations**

Investigators are responsible for monitoring the safety of patients who have entered this study and for alerting Lilly or its designee to any event that seems unusual, even if this event may be considered an unanticipated benefit to the patient.

The investigator is responsible for the appropriate medical care of patients during the study.

The investigator remains responsible for following, through an appropriate health care option, adverse events that are serious or that caused the patient to discontinue before completing the study. The patient should be followed until the event is resolved or explained. Frequency of follow-up evaluation is left to the discretion of the investigator.

***10.3.1. Adverse Events***

Lilly has standards for reporting adverse events that are to be followed regardless of applicable regulatory requirements that may be less stringent. A clinical trial adverse event is any untoward medical event associated with the use of a drug or drug delivery system in humans, whether or not it is considered related to a drug or drug delivery system.

Lack of drug effect is not an adverse event in clinical trials because the purpose of the clinical trial is to establish drug effect.

Cases of pregnancy that occur during maternal or paternal exposures to study drug or drug delivery system should be reported. Data on fetal outcome and breast-feeding are collected for regulatory reporting and drug safety evaluation.

Study site personnel will record the occurrence and nature of each patient’s preexisting conditions, including clinically significant signs and symptoms of the disease under

treatment in the study.

During the study, site personnel will record any change in the condition(s) and the occurrence and nature of any adverse events.

After the Informed Consent Document (ICD) is signed, site personnel will record any change in the condition(s) and the occurrence and nature of any AEs. All AEs related to protocol procedures are reported to Lilly or designee via electronic data entry.

In addition, **all** adverse events occurring after the patient receives the first dose of study drug must be reported to Lilly or its via designee electronic data entry.

Investigators will be instructed to report to Lilly or its designee their assessment of the potential relatedness of each adverse event to protocol procedure or study drug, and/or drug delivery system via electronic data entry.

Study site personnel must alert Lilly or its designee within 24 hours of the investigator

**unblinding** a patient’s treatment group assignment for any reason.

If a patient's dosage is reduced or treatment is discontinued as a result of an adverse event, study site personnel must clearly report to Lilly or its designee via electronic data entry the circumstances and data leading to any such dosage reduction or discontinuation of treatment.

Any clinically significant findings from ECGs, labs, or vital sign measurements that result in a diagnosis should be reported to Lilly or its designee using the “Adverse Events/Preexisting Condition” page of the electronic data entry.

**10.3.1.1. Serious Adverse Events (SAEs)**

SAE collection begins after the patient has signed informed consent and has received study drug. If a patient experiences an SAE after signing informed consent, but prior to receiving study drug, the event will NOT be collected unless the investigator feels the event may have been caused by a protocol procedure.

Study site personnel must alert Lilly or its designee of any **serious** adverse event (SAE) within 24 hours of investigator awareness of the event via a Sponsor-approved method. Alerts issued via telephone are to be immediately followed with official notification on study-specific SAE forms. An SAE is any adverse event from this study that results in one of the following outcomes:

• death

• initial or prolonged inpatient hospitalization

• a life-threatening experience (that is, immediate risk of dying)

• persistent or significant disability/incapacity

• congenital anomaly/birth defect

• considered significant by the investigator for any other reason.

Important medical events that may not result in death, be life-threatening, or require hospitalization may be considered serious adverse drug events when, based upon appropriate medical judgment, they may jeopardize the patient or subject and may require medical or surgical intervention to prevent one of the outcomes listed in this definition.

Lilly or its designee will be alerted to SAEs occurring within 30 days of a patient’s discontinuation, regardless of the investigator’s opinion of causation. Thereafter, serious adverse events are not required to be reported unless the investigator feels the events

were related to either study drug, or drug delivery system, or a protocol procedure.

***10.3.2. Other Safety Measures***

Twelve-lead ECGs will be obtained according to the Study Schedule (Protocol

Attachment LYDZ(b).1).

The ECGs will be interpreted by a qualified physician (the investigator or qualified designee) at the site as soon after the time of ECG collection as possible, and ideally while the patient is still present, for immediate patient management and to determine whether the patient meets entry criteria. If a clinically significant increase in the QTc interval from baseline is present, then the investigator should assess the patient for symptoms (such as palpitations, near syncope, syncope). See Protocol Attachment LYDZ(b).4 Adult Electrocardiogram Criteria.

The ECGs will subsequently be electronically transmitted via modem to the centralized ECG vendor designated by Lilly. The centralized ECG vendor’s cardiologist will then complete the ECG overread. The central ECG vendor’s overread will be used for data analysis and report writing purposes.

Once the overread ECG is returned from the centralized ECG vendor, the investigator or qualified designee is responsible for determining if any change to the patient management is needed and must document his/her review.

If there are differences in ECG interpretation between the investigator or qualified designee and the ECG vendor cardiologist, the investigator or qualified designee's interpretation will prevail for study entry and immediate patient management purposes, and the ECG vendor cardiologist's will prevail for data analysis purposes.

***10.3.3. Safety Monitoring***

The Lilly clinical research physician will monitor safety data throughout the course of the study.

Lilly will review SAEs within time frames mandated by company procedures and will review trends, laboratory analytes, and adverse events at periodic intervals.

In the event that safety monitoring uncovers an issue that needs to be addressed by unblinding at the group level, only members of the data monitoring board (an advisory group for this study formed to protect the integrity of data; refer to Section 12.2.11) can conduct additional analyses of the safety data.

***10.3.4. Complaint Handling***

Lilly collects product complaints on study drugs and drug delivery systems used in clinical trials in order to ensure the safety of study participants, monitor quality, and to facilitate process and product improvements.

Complaints related to unblinded comparator drugs or concomitant drugs/drug delivery systems are reported directly to the manufacturers of those drugs/devices in accordance with the package insert.

For blinded studies, all product complaints associated with material packaged, labeled, and released by Lilly will be reported via product complaint forms.

The investigator or his/her designee is responsible for handling the following aspects of the product complaint process in accordance with the instructions provided for this study:

• recording a complete description of the product complaint reported and any associated adverse events using the study-specific complaint forms provided for this purpose

• faxing the completed product complaint form within 24 hours to Lilly or its designee.

If the investigator is asked to return the product for investigation, he/she will return a copy of the product complaint form with the product.

**10.4. Sample Collection and Testing**

Protocol Attachment LYDZ(b).3 lists the specific tests performed for this study.

***10.4.1. Samples for Standard Laboratory Testing***

Blood and urine samples will be collected at the times specified in the Study Schedule (Protocol Attachment LYDZ(b).1). Standard laboratory tests, including chemistry, hematology, and urinalysis panels will be performed. A pregnancy test will be performed (if applicable). All clinical laboratory tests will be analyzed by a central laboratory.

Protocol Attachment LYDZ(b).2 lists the specific tests that will be performed for this study.

Atomoxetine is metabolized through the CYP2D6 metabolic pathway, which is polymorphic in humans, and the magnitude of plasma exposure varies with CYP2D6 genotype. Both extensive metabolizer (EM) and poor metabolizer (PM) patients were studied during the clinical development program, and no serious safety concerns have been identified in either group at atomoxetine doses up to 160 mg/day.

A blood sample may be drawn assayed for cytochrome P450 2D6 (CYP2D6) genotype per investigator request in the event of a clinically significant overdose, serious adverse event (SAE), or if a patient is found to have a new onset of neurological signs and symptom. The Lilly Study Physician must be contacted for approval to draw and analyze the sample. The investigator will receive the CYP2D6 genotype results.

Investigators must document their review of each laboratory report.

Samples collected for specified laboratory tests will be destroyed within 60 days of receipt of confirmed test results. Certain samples may be retained for a longer period, if necessary, to comply with applicable laws, regulations, or laboratory certification standards.

**10.5. Appropriateness of Measurements**

The objective of this study is to evaluate the effectiveness of atomoxetine in young adult patients who have clinically significant ADHD. The CAARS–Inv-SV assesses core symptoms of ADHD and has been used as the primary instrument in previous studies of atomoxetine in ADHD adults (B4Z-MC-LYAA/AO/AR/BM). Its psychometric properties are well-established. The CGI-ADHD-S provides a global assessment. The MADRS is widely used and well-validated instruments for assessing depression. The AAQOL is the only disease specific quality of life instrument available for adult ADHD. The BAI is a widely used and well-validated instrument for assessing anxiety. The additional various functional outcome measures have been broadly used in various trials with multiple disease states to study functional outcomes in patients with various psychiatric conditions.

**11. Data Quality Assurance**

To ensure accurate, complete, and reliable data, Lilly or its representatives will do the following:

• provide instructional material to the study sites, as appropriate.

• sponsor a start-up training session to instruct the investigators and study coordinators. This session will give instruction on the protocol, the completion of the e-CRFs, and study procedures.

• make periodic visits to the study site.

• be available for consultation and stay in contact with the study site personnel by mail, telephone, and/or fax.

• review and evaluate e-CRF data and use standard computer edits to detect errors in data collection.

• conduct a quality review of the database.

In addition, Lilly or its representatives may periodically check a sample of the patient data recorded against source documents at the study site. The study may be audited by Lilly Medical Quality Assurance (MQA) or its representatives, and/or regulatory agencies at any time. Investigators will be given notice before an audit occurs.

To ensure the safety of participants in the study, and to ensure accurate, complete, and reliable data, the investigator will keep records of laboratory tests, clinical notes, and patient medical records in the patient files as original source documents for the study. If requested, the investigator will provide the sponsor, applicable regulatory agencies, and applicable ethical review boards (ERBs) with direct access to original source documents.

**11.1. Direct Data Entry and Computerized Systems**

An electronic data capture system will be used in this trial. Some or all of a patient’s data (for example, a rating scale, daily dosing schedule, patient diary) may be directly entered into the system on an eCRF (for example, on a web-based data entry screen) at the time that the information is obtained. In these instances where there is no prior written or electronic record of the data, the eCRF will serve as the source document.

The e-CRF data collected by the third party vendor will be encoded by the third party vendor and stored electronically in the third party vendor’s database system. Validated data will subsequently be transferred to the sponsor’s data warehouse, using standard Lilly file transfer processes.

Data managed by a central vendor, such as laboratory test data or ECG data, will be stored electronically in the central vendor’s database system. Data will subsequently be transferred from the central vendor to the Lilly generic labs system.

Any data for which the e-CRF will serve as the source document will be identified and documented by each site in that site’s study file.

Data from complaint forms submitted to Lilly will be encoded and stored in the global product complaint management system.

**12. Sample Size and Statistical Methods**

**12.1. Determination of Sample Size**

The primary objective for Study LYDZ(b) is to compare the efficacy improvement from baseline of atomoxetine dosed twice daily (BID) compared with placebo after 12 weeks in young adults using the change from baseline in the Conners’ Adult ADHD Rating Scale-Investigator Rated: Screening Version (CAARS-Inv:SV) total ADHD symptom score, the primary efficacy measure.

Approximately 440 patients will be randomized in a 1:1 ratio between atomoxetine and placebo (220 patients per group). Based on a similar ongoing trial (LYDQ) it is assumed that approximately 68% of the enrolled patients will complete the study (12 weeks of therapy). Studies LYAA & LYAO had drop-out rates of 25% & 33%, respectively, following 10 weeks of therapy.

With 220 patients per arm, assuming a 68% completion rate and the same covariance as observed in study LYCU, which includes a residual variance of 40 at week 16, and an estimated effect size of atomoxetine over placebo of 0.35, using a 5% significance level, the primary MMRM analysis is expected to have 90% power to detect a difference between atomoxetine and placebo at week 12. Two previous placebo-controlled adult ADHD atomoxetine studies (B4Z-MC-LYAA and B4Z-MC-LYAO) demonstrated

similar effect sizes on a variation of this scale, the CAARS-Inv:SV total ADHD symptom

score. The effect sizes in these two trials, based on repeated measures, were 0.35 and 0.4 (Michelson et al 2003). This sample size has been selected to achieve similar effects and allow for more than 80% power at the end of the study, assuming a 68% retention rate over 12 weeks. The sample size was estimated using the repeated measures tool

available via the Lilly Statistics website. The following table lists the required sample sizes for various effect sizes and two different drop-out rates, assuming 90% power.

**Table LYDZ(b).2. Total N Required for Various Combinations of Effect Size and Drop-out Rate**

|  | Early Drop-out Rate | |
| --- | --- | --- |
| Effect Size | 32% | 50% |
| 0.3 | 606 | 752 |
| 0.35 | 440 | 554 |
| 0.4 | 340 | 422 |

This result is nearly identical to that obtained from the last-observation-carried-forward (LOCF) approach for a two-sided test at the 5% significance level, with an assumed standard deviation of 10.0 and 85% power to detect a difference of 3.5 points in the mean change of the CAARS-Inv:SV total ADHD symptom score. These assumptions were

based on the results from the 18-25 year old patients in LYAA & LYAO and the 18-30 year old patients in LYCU.

This sample size will also allow for an effect size of 0.325 with 80% power for the first gated secondary outcome of the AAQOL-29 total score. If the effect size increases to

0.35 or 0.40, the power will increase to 85% or 93%, respectively. The observed effect size for 18-30 year old patients in LYCU was 0.41.

The Fagerstrom Test for Nicotine Dependence, Timeline Followback: Habits, BRIEF-A, Epworth Sleepiness Scale, SASS, and PGI-I scales have never been collected in any randomized double-blind atomoxetine adult trial, so no formal power computation is possible.

These LOCF sample sizes were calculated using the "Two group t-test of equal means

(equal n’s)" module of nQuery Advisor 4.0 (1995-2000, Janet D. Elashoff).

**12.2. Statistical and Analytical Plans**

***12.2.1. General Considerations***

Statistical analysis of this study will be the responsibility of Eli Lilly and Company or an approved vendor of Eli Lilly and Company.

Treatment effects for all efficacy and safety variables will be evaluated based on a two- sided significance level of 0.05, and the interaction effects at 0.10. Any change to the data analysis methods described in the protocol will require an amendment ONLY if it changes a principal feature of the protocol. Any other changes in the data analysis methods described in this protocol will be documented in the Statistical Analysis Plan (SAP) prior to unblinding of the data. Such changes will not require protocol amendments. If changes to the data analysis methods described in the protocol are deemed necessary the justification for making such changes will be described in the clinical study report. Additional exploratory analyses of the data will be conducted as deemed appropriate.

Active treatment phase refers to the visit interval after randomization in which the placebo lead-in period is excluded. In all the analyses, baseline will be the latest non- missing observation across all the visits in the screening phase and the placebo lead-in period, before the active study drug begins. Endpoint will be the latest non-missing observation across all the post-baseline visits before.

**12.2.1.1. Analysis Populations**

Two populations will be considered for analysis in this protocol, one based on the intent- to-treat principle and the other is based on patients who have received at least 1 dose of study medication. For all efficacy analyses, an intent-to-treat population will be analyzed. This population consists of all patients randomized to a treatment group, even

if the patient did not take the assigned treatment, did not receive the correct treatment, or did not otherwise follow the protocol. All enrolled patients who had both a baseline and at least 1 post baseline score will be included in the analysis of the questionnaire data.

All patients who take at least 1 dose of study medication will be included in the analysis of safety data. It will be assumed that patients who receive medication during Study Period II have taken at least 1 dose, unless it is documented otherwise.

The following investigator pooling algorithm will apply to the primary and all other analyses – investigative sites with less than 2 patients per treatment arm will be pooled with the next smallest investigative site until that pooled site has at least 2 patients per

treatment arm.

**12.2.1.2. Adjustment for Multiplicity**

All tests of significance in this study will be conducted at a 2-sided alpha level of 0.05, unless otherwise stated. The primary efficacy analysis will be performed on the original scale data. All other analyses will also be performed on the original scale data. No adjustments for multiple comparisons will be made. All tests of hypotheses will be considered statistically significant if the two-sided p-value is less than 0.05. No adjustments for secondary comparisons are needed since we are using a gatekeeper strategy to prevent multiplicity in the comparisons that are of the greatest interest.

***12.2.2. Patient Disposition***

The percentage of patients who discontinue for various reasons (that is, lack of efficacy, adverse event) and the percentage of patients who complete the double-blind period among all randomized patients will be calculated for Study Period II. The percentages of discontinuations for each reason will be compared between treatment groups using Fisher’s exact test.

***12.2.3. Patient Characteristics***

Patient characteristics (demographics and baseline psychiatric measures) will be summarized for all entered as well as all randomized patients. For randomized patients, comparisons of patient characteristics will be made across treatment groups using Fisher’s exact test for categorical data and using an analysis of variance (ANOVA) with treatment as an independent effect for quantitative data.

***12.2.4. Concomitant Therapy***

Concomitant medications during Study Period II will be tabulated and compared across treatment groups using Fisher’s exact test.

***12.2.5. Treatment Compliance***

Compliance will be tabulated at each visit during Study Period II. The percentage of patients who are compliant at all visits during Study Period II will be tabulated and compared across treatment groups using Fisher’s exact test.

***12.2.6. Primary Outcome and Methodology***

The primary summary measure, the CAARS-Inv:SV total ADHD symptom score as well as the Inattention subscale score, and Hyperactivity-Impulsivity subscale score, will be analyzed using a restricted maximum likelihood (REML)-based, mixed model repeated measures (MMRM) technique. The model will include the fixed, categorical effects of treatment, investigator, visit, and treatment-by-visit interaction as well as the continuous, fixed covariates of baseline (last of scores at Visit 1 and Visit 2) score and baseline score- by-visit interaction. The following covariance structures will be fit to the model: unstructured, heterogeneous toeplitz, heterogeneous autoregressive, heterogeneous compound symmetry and simple. The correlation structure converging to the best fit as measured by Akaike's Information Criterion will be used as the primary analysis. The Kenward-Roger approximation will be used to estimate denominator degrees of freedom. Significance tests will be based on least-squares means and Type III sum-of-squares. Analyses will be implemented using SAS PROC MIXED. The primary comparison is

the contrast between treatments at Visit 6 (12 weeks post-baseline). Secondary evidence of efficacy from the primary analysis will be based on the main effect of treatment and

the treatment-by-visit interaction terms from the MMRM analysis. This measure will be summarized separately during Study Period III. All statistical tests will be two-sided and will be conducted at the 5% significance level.

***12.2.7. Secondary Analyses of the Primary Outcome***

As supporting evidence, the CAARS-Inv:SV total ADHD symptom score as well as the Inattention subscale score, and Hyperactivity-Impulsivity subscale score, will be analyzed using a last observation carried forward (LOCF) analysis. Change from baseline to LOCF endpoint will be compared across treatments using an ANCOVA model with terms for baseline, treatment, and investigator.

***12.2.8. Gated Secondary Efficacy Analyses***

The first gated secondary objective is to assess the efficacy of atomoxetine therapy compared with placebo on the total score of the Adult ADHD Quality of Life -29 (AAQOL-29) using a treatment contrast from the repeated measures mixed effects model at the final post baseline visit. The repeated measures model will include the same terms as that used for the primary efficacy measure. This measure will be summarized separately during Study Period III.

The next gated secondary objective is to assess the efficacy of atomoxetine therapy compared with placebo on the subscales of the AAQOL-29 in the following order: Relationship; Life Productivity; Psychological Health; and Life Outlook using a treatment contrast from the repeated measures mixed effects model at the final post baseline visit. The repeated measures model will include the same terms as that used for the primary efficacy measure. These subscale measures will be summarized separately during Study Period III.

**12.2.8.1. Secondary Efficacy Analyses**

The secondary efficacy analyses will be conducted for the variables included in, but not limited to, those in Table LYDZ(b).3.

**Table LYDZ(b).3. Description of Secondary Efficacy Variables**

Analysis Variable Description

1. All post-baseline scores for:

a. CAARS-S:SV total ADHD symptom score and subscores

2. Change from baseline to endpoint for the following Total scores and subscores:

a. AAQOL-29

b. CAARS-S-SV

d. PGI-I

e. Habits Timeline Followback: (Alcohol, Nicotine, Caffeine, Marijuana, Drug)

f. Fagerstrom Test for Nicotine Dependence g. SASS

h. Driving Behavior Survey-S i. Driving Behavior Survey-O j. BRIEF-A

k. Epworth Sleepiness Scale l. Beck Anxiety Inventory

m. CGI-ADHD-S

n. MADRS

Post-baseline during Study Period II is defined as

Visits 3-6.

Difference between baseline and last observation on study drug (LOCF change scores). See notes on computations of total and subscores below.

3. Indicator variable for responders 3. Patients will be classified as responders according to 2 definitions:

a. 25% or greater decrease in ADHD symptoms as measured by the CAARS-Inv-SV total ADHD symptom score OR

b. Decrease on CGI-ADHD-S of 2 or more

4. Indicator variable for strong responders 3. Patients will be classified as strong responders according to the following definition:

a. 40% or greater decrease in ADHD symptoms as measured by the CAARS-Inv-SV Total

ADHD symptom score

5. Indicator variable for remitters 4. Patients will be classified as remitters according to the following definition:

a. Endpoint CGI-ADHD-S = 1 or 2

6. Indicator variable for drug compliance 5. Compliance rates will be compared across treatment groups overall and by visit.

Abbreviations: ADHD = Attention-Deficit/Hyperactivity Disorder; CAARS-Inv-SV = Conners’ Adult ADHD Rating Scale-Investigator Rated: Screening Version; CAARS-S-SV = Conners’ Adult ADHD Rating Scale-Self Rated: Screening Version; PGI-I = Patient Global Impression-Improvement; SASS

= Social Adaptation Self-Evaluation Scale; Driving Behavior Survey (Self-Report); Driving Behavior Survey (Other-Report); BRIEF-A = Behavior Rating Inventory of Executive Function-Adult Version; CGI-ADHD-S = Clinical Global Impressions-ADHD-Severity; MADRS = Montgomery-Asberg Depression Rating Scale ; LOCF = last observation carried forward.

Variables 1-5 will be summarized for Study Period II using the last of Visits 1 and 2 as baseline and Visits 3-6 as the post-baseline visits. Variable 1a will be analyzed during Study Period II using a restricted maximum likelihood (REML)-based mixed effects model, repeated measures technique as defined for the primary efficacy analysis. This variable will be analyzed over the course of Study Period II using a contrast from the repeated measures mixed effects model at the final post baseline visit. Additionally,

visit-wise efficacy will be assessed using contrasts from this model at each post baseline

visit. This variable will be summarized separately by visit during Study Period III.

Variables 2.a through 2.n will be analyzed using a fixed-effects analysis of covariance (ANCOVA) model with terms for baseline score, treatment, and investigator. These variables will be summarized separately during Study Period III.

Variables 3-6 will be compared across treatment groups during Study Period II and separately during Study Period III using a Fisher’s Exact Test.

Time to response and time to remission will be compared across treatment groups during Study Period II using the Kaplan-Meier Product limit estimator, with the test for treatment difference being based on the log-rank test.

Correlation between the AAQOL-29 total score and the CAARS-Inv:SV total ADHD symptom score will be computed using Pearson’s correlation co-efficient to assess the relationship between these two measures.

A Fisher’s Exact Test will be used to assess baseline smoking status as a predictor of response (a 25% decrease from baseline on the CAARS-Inv:SV total ADHD symptom score) and strong response (a 40% decrease from baseline on the CAARS-Inv:SV total ADHD symptom score ) to atomoxetine treatment compared with placebo. If sparse data exists, a Chi-Squared analysis may be used instead.

***12.2.9. Safety Analyses***

Safety analysis will include all randomized patients who take at least one dose of study drug.

The variables included in the safety analysis will include, but not necessarily be restricted to, those listed in Table LYDZ(b).4.

Safety will be assessed by summarizing and analyzing adverse events, vital signs, weight, ECGs, and concomitant medications use.

**Table LYDZ(b).4. Description of Safety Variables**

Analysis Variable Description

1.Change from baseline to last observation:

a. Vital signs and weight b. ECGs

2. Indicator variable for:

a. Serious adverse events

b. Discontinuations due to adverse events c. Treatment-emergent adverse events

1. Difference between baseline and last observation on study drug (LOCF change scores). Only patients having a baseline and at least one post-baseline measure that have taken at least 1 dose of study drug will be included in the analysis. a. N/A

b. ECG interval times and heart rate QTc intervals will be analyzed using the Fredericia’s correction factor and a data-based correction factor.

2.

a-b. N/A

c. A treatment-emergent adverse event is an adverse event that first occurred or worsened post- baseline.

3. Concomitant medications 3. N/A

4. Dosing 4. Mean, final and modal dose

5. Exposure 5. Summarized by visit

Variables 1.a-1.b will be compared across treatment groups using a fixed effects ANCOVA model incorporating treatment and investigator effects and baseline as a covariate using the raw observation as the dependent variable. Variables 2.a-2.c and 3 will be analyzed by comparing frequencies for each treatment group using Fisher’s Exact test. Baseline for determining treatment emergence will be defined as Visit 1 through Visit 2. The treatment period for determining treatment emergence will be defined as Visit 3 through Visit 6.

Mean, modal, and final doses will be tabulated for the atomoxetine arm. Exposure to study drug will be tabulated for both treatment groups.

Categorical analyses of vital signs and ECGs will be performed using the clinically significant thresholds defined in the atomoxetine integrated summary of safety.

Time to discontinuation will be compared across treatment groups in a similar manner to that used for time to response and time to remission.

These safety measures will be summarized separately during Study Period III.

***12.2.10. Subgroup Analyses***

The subgroup analyses will include analyses between each subgroup of patients as well as statistical models that test for subgroup effects and treatment by subgroup interaction.

Subgroups of patients for efficacy analyses will include investigational site, prior- stimulant-use strata, ADHD subtype, gender, and age.

For investigational site subgroup analyses (pooled as described in Section 12.2.1.1), changes in CAARS-Inv-SV total ADHD symptom score will be assessed using an ANCOVA model, including terms for baseline, treatment, subgroup, and the treatment- by-subgroup interaction. If the interaction is statistically significant at the 0.10 level, then the potential causes of the interaction will be investigated.

The incidence of treatment-emergent adverse events (TEAE) will be summarized by the following subgroups; investigational site, prior-stimulant-use strata, ADHD subtype, gender, and age.

***12.2.11. Interim Analyses***

No interim analyses are planned for this study. If an unplanned interim analysis is deemed necessary, the appropriate Lilly regulatory scientist will be consulted to determine whether it is necessary to amend the protocol.

Only the data monitoring committee (DMC) is authorized to review completely unblinded interim efficacy and safety analyses. Study sites will receive information about interim results ONLY if they need to know for the safety of their patients.

At the end of Study Period III, all data will be brought in house and locked. This will not be considered an interim analysis since this is considered the final lock of the study.

Study Period II data will be analyzed separately from Study Period III, the open label portion of this trial. It may be possible for the CS to continue after the final lock has occurred for this trial. This will not impact the final lock on the CS as this is completely separate from the trial and will not impact the planned analyses.

**13. Informed Consent, Ethical Review, and Regulatory**

**Considerations**

**13.1. Informed Consent**

For this study, privacy statement/opt-in consents and informed consents are obtained and electronically signed online via the study website. Prior to the first level of screening (completion of ASRS scale to determine likelihood of meeting ADHD criteria), a waiver with a privacy statement/opt-in consent is obtained. If the subject has a positive score on the ASRS at the first level of screening, they must opt-in via the study website prior to completing questions regarding inclusion/exclusion criteria (the second level of screening for the study). This opt-in statement will provide a brief overview of the RCT and CS,

though the subject will not electronically sign the consent for the RCT or CS until after completing the inclusion/exclusion criteria survey. After completion of the inclusion/exclusion criteria questions, the subject will be informed of eligibility for the RCT. If the subject is eligible for and chooses to participate in the RCT, an informed consent will be obtained at the investigator site by qualified site personnel. Similarly, if the subject opts-in for the CS, an online self-administered informed consent will be

obtained prior to entry into the CS.

Both the RCT and CS in this study utilize the rating of an “observer” identified by the patient. A self-administered informed consent will be obtained online via the study website from the person identified as the observer to complete the driving behavior rating scale. Additionally, the observer will do all driving behavior ratings through the study website.

For the RCT, the investigator is responsible for ensuring that the patient understands the risks and benefits of participating in the study, including answering any questions the patient may have throughout the study and sharing in a timely manner any new information that may be relevant to the patient's willingness to continue his or her participation in the trial.

The informed consent document will be used to explain the risks and benefits of study participation to the patient in simple terms before the patient is entered into the study, and to document that the patient is satisfied with his or her understanding of the risks and benefits of participating in the study and desires to participate in the study.

The investigator is responsible for ensuring that informed consent is given by each patient. This includes obtaining the appropriate signatures on the ICD prior to the performance of any protocol procedures and prior to the administration of study drug.

**13.2. Ethical Review**

Lilly must agree with all ICDs before they are submitted to the ERB and are used at investigative sites(s). All informed consent documents must be compliant with the

International Conference on Harmonization (ICH) guideline on good clinical practice (GCP). Informed consent obtained under special circumstances may occur only if allowed by local laws and regulations and performed in accordance with a written process approved by Lilly.

Documentation of ERB approval of the protocol and the ICD must be provided to Lilly *before* the study may begin at the investigative site(s). The ERB(s) will review the protocol as required.

Any member of the ERB who is directly affiliated with this study as an investigator or as site personnel must abstain from the ERB’s vote on the approval of the protocol.

The study site’s ERB should be provided with the following:

• the current Investigator’s Brochure or package labeling and updates during the course of the study

• Informed Consent Document

• relevant curricula vitae.

**13.3. Regulatory Considerations**

This study will be conducted in accordance with the ethical principles that have their origin in the Declaration of Helsinki and that are consistent with good clinical practices and the applicable laws and regulations. The investigator, head of the medical institution, or designee will promptly submit the protocol to applicable ERB(s).

Atomoxetine is being studied in the United States (US) under a US Investigational New

Drug (IND) application. The US IND number is 19,228.

All or some of the obligations of the sponsor may be assigned to a contract research organization (CRO).

An identification code assigned by the electronic data capture system to each patient will be used in lieu of the patient's name to protect the patient's identity when reporting adverse events and/or other trial-related data.

***13.3.1. Investigator Information***

Board-certified or eligible physicians (MD or DO) with clinical research experience who are qualified to evaluate and treat adult ADHD patients will participate as investigators in this clinical study. Clinical psychologists with similar research and clinical skills can

also serve as a principal investigator provided he/she works with a sub-investigator who

is a qualified physician (MD or DO).

***13.3.2. Protocol Signatures***

After reading the protocol, each principal investigator will sign the protocol signature page and send a copy of the signed page to a Lilly representative.

***13.3.3. Final Report Signature***

The sponsor’s responsible medical officer will sign the final clinical study report for this study, confirming that, to the best of his or her knowledge, the report accurately describes the conduct and results of the study.

**14. References**

APA [American Psychiatric Association]. 2000. Diagnostic and statistical manual of mental disorders. 4th ed. Text revised. Washington, DC: American Psychiatric Assoc

B4Z-MC-LYBV: A Double-Blind Study of Functional Outcomes with Atomoxetine Hydrochloride and Placebo in Adult Outpatients with DSM-IV Attention-Deficit/ Hyperactivity Disorder. Eli Lilly and Company. Data on File.

B4Z-US-LYCU: Efficacy and Safety of Once-Daily Atomoxetine Hydrochloride in Adults with ADHD over an Extended Period of Time (6 months): With a Brief Evaluation of Executive Cognition. Eli Lilly and Company. Data on File.

Barkley R. 1998. Attention-Deficit Hyperactivity Disorder: A Handbook for Diagnosis and Treatment. p 82-85.

Barkley RA, Murphy KR, Kwasnik D. 1996. Motor vehicle driving competencies and risks in teens and young adults with attention deficit hyperactivity disorder. Pediatrics

98(6 Pt 1):1089-1095.

Biederman J, Faraone SV, Spencer T, Wilens T, Norman D, Lapey KA, Mick E, Lehman BK, Doyle A. 1993. Patterns of psychiatric comorbidity, cognition, and psychosocial functioning in adults with attention deficit hyperactivity disorder. Am J Psychiatry

150:(12)1792-1798.

Biederman J, Monuteaux MC, Mick E, Spencer T, Wilens TE, Silva JM, Snyder LE, Faraone SV. 2006. Young adult outcome of attention deficit hyperactivity disorder: a controlled 10-year follow-up study. Psychol Med 36(2):167-179.

Bosc M, Dubini A, Polin V. 1997. Development and validation of a social functioning scale, the Social Adaptation Self-evaluation Scale. Eur Neuropsychopharmacol 7 (Suppl 1):S57-S70.

Brod M, Perwien A, Adler L, Spencer T, Johnston J. 2005. Conceptual Model for

Measuring Functional Impairments in Adults with ADHD. Prim Psychiatry 12(6):58-

64.

Conners CK, Erhardt D, Sparrow E. 1999. Conners' Adult ADHD Rating Scales

(CAARS). North Tonawanda, NY: Multi-Health Systems Inc.

Guy W. 1976. ECDEU assessment manual for psychopharmacology, revised 1976.

National Institute of Mental Health, Psychopharmacology Research Branch Rockville

MD 217-222-313-331.

Hechtman L. 1992. Long-term outcome in attention-deficit hyperactivity disorder. Child and Adolesc Psychiatr Clin N Am 1:553-565.

Jadad AR, Boyle M, Cunningham C, Kim M, Schachar R. 1999. Treatment of Attention- Deficit/Hyperactivity Disorder. Evid Rep Technol Assess (Summ) No. 11 (Prepared by McMaster University under Contract No. 290-97-0017). AHRQ Publication No 00- E005 Rockville, MD: Agency for Healthcare Research and Quality.

Johns MW. 1991. A new method for measuring daytime sleepiness: the Epworth sleepiness scale. Sleep 14(6):540-545.

Kessler RC, Adler L, Barkley R, Biederman J, Conners CK, Demler O, Faraone SV, Greenhill LL, Howes MJ, Secnik K, Spencer T, Ustun TB, Walters EE, Zaslavsky AM.

2006. The prevalence and correlates of adult ADHD in the United States: results from the National Comorbidity Survey Replication. Am J Psychiatry 163(4):716-723.

Mahone EM, Cirino PT, Cutting LE, Cerrone PM, Hagelthorn KM, Hiemenz JR, Singer HS, Denckla MB. 2002. Validity of the behavior rating inventory of executive function in children with ADHD and/or Tourette syndrome. Arch Clin Neuropsychol 17(7):643-

662.

McCabe SE, Knight JR, Teter CJ, Weschler H. 2005. Non-medical use of prescription stimulants among US college students: prevalence and correlates from a national survey. Addiction 100:96-106.

McCabe SE, Teter CJ, Boyd CJ. 2006. Medical use, illicit use and diversion of prescription stimulant medication. J Psychoactive Drugs 38:43-56.

Michelson D, Adler L, Spencer T, Reimherr FW, West SA, Allen AJ, Kelsey D, Wernicke J, Dietrich A, Milton D. 2003. Atomoxetine in adults with ADHD: two randomized, placebo-controlled studies. Biol Psychiatry 53(2):112-120.

Montgomery SA, Asberg M. 1979. A new depression scale designed to be sensitive to change. Br J Psychiatry 134:382-389.

NIMH. 1976. ECDEU assessment manual for psychopharmacology, revised 1976.

Rockville, MD: National Institute of Mental Health, Psychopharmacology Research

Branch 217-222-313-331.

Novak S, Kroutil LA, Williams RL, Van Brunt D. 2006. NonMedical Use and Diversion of ADHD Medication in the United States. Data on File - Eli Lilly.

Pomerleau OF, Downey KK, Stelson FW, Pomerleau CS. 1995. Cigarette smoking in adult patients diagnosed with attention deficity hyperactivity disorder. J Subst Abuse

7(3):373-378.

Rothwell PM. 2005. External validity of randomized controlled trials: "to whom do the results of this trial apply?". Lancet 365(9453):82-93.

Sobell L, Sobell M. 1992. Timeline Follow Back: a technique for assessing self-reported alcohol consumption. Psychological and Biological Methods 41-72.

Sobell L, Sobell M. 1996. Timeline Followback User Manual.

Trivedi MH, Rush H. 1994. Does a placebo run-in or a placebo treatment cell affect the efficacy of antidepressant medications? Neuropsychopharmacology 11:33-43.

Westfall P. 2001. Gatekeeping Strategies for Testing Primary and Secondary Endpoints.

Drug Information Association Meeting March 2001.

**Protocol Attachment LYDZ(b).1.**

**Study Schedule**

**Study Schedule, Protocol B4Z-US-LYDZ(b)**

| **Study Period** | **I** | | **II** | | | | **III** | | |
| --- | --- | --- | --- | --- | --- | --- | --- | --- | --- |
| **Visit** | **1** | **2** | **3** | **4** | **5** | **6**  **SUM IIa** | **7** | **8** | **9**  **SUM IIIa** |
| **Weeks Until Next Visit:** | **1** | **2** | **3** | **3** | **4** | **2** | **5** | **5** | **N/A** |
| **Interval Between Visits and**  **Target ( )** | **5-28 (7)** | **10-18 (14)** | **17-25 (21)** | **17-25 (21)** | **25-31 (28)** | **10-18 (14)** | **28-38 (35)** | **28-38 (35)** | **-** |
| Informed consentb | X |  |  |  |  |  |  |  |  |
| Patient number assigned | X |  |  |  |  |  |  |  |  |
| Inclusion/exclusion criteria  (including PHQ-9) | X |  |  |  |  |  |  |  |  |
| Presenting/preexisting conditionsc | X |  |  |  |  |  |  |  |  |
| Medical/psychiatric history | X |  |  |  |  |  |  |  |  |
| Physical Exam | X |  |  |  |  |  |  |  |  |
| Height | X |  |  |  |  |  |  |  |  |
| Weight | X | X | X | X | X | X | X | X | X |
| Vital signs | X | X | X | X | X | X | X | X | X |
| Demographics | X |  |  |  |  |  |  |  |  |
| Social Demographics | X |  |  |  |  |  |  |  |  |
| Previous drug therapy- ADHD | X |  |  |  |  |  |  |  |  |
| Previous drug therapy- all other  CNS Therapies | X |  |  |  |  |  |  |  |  |
| Concomitant medications | X | X | X | X | X | X | X | X | X |
| Adverse events | X | X | X | X | X | X | X | X | X |
| Dispense drug |  | X | X | X | X | X | X | X |  |
| Study drug compliance |  |  | X | X | X | X | X | X | X |
| **Investigator-Administered Scales:** | | | | | | | | | |
| ASRS (prior to v1 via web) | X |  |  |  |  |  |  |  |  |
| SCID-RV | X |  |  |  |  |  |  |  |  |
| ACDS v1.2 | X |  |  |  |  |  |  |  |  |
| CAARS-Inv-SV | X | X | X | X | X | X | X | X | X |
| CGI-ADHD-S | X | X | X | X | X | X | X | X | X |
| MADRS | X |  |  | X |  | X |  |  | X |
| **Patient-Rated Scales: d** | | | | | | | | | |
| AAQOL-29 |  | X | X | X | X | X |  |  | X |
| CAARS-S:SV | X | X | X | X | X | X | X | X | X |
| PGI-I |  |  | X | X | X | X |  |  | X |
| Habits Timeline Followback: (Alcohol, Nicotine, Caffeine, Marijuana, and Drug) |  | X | X | X | X | X |  |  | X |
| Fagerstrom Test for Nicotine  Dependence |  | X |  | X |  | X |  |  | X |
| SASS |  | X |  | X |  | X |  |  |  |
| Driving Behavior Survey - Self  Report |  | X |  | X |  | X |  |  |  |

|  |  | |  | | | |  | | |
| --- | --- | --- | --- | --- | --- | --- | --- | --- | --- |
| **Study Period** | **I** | | **II** | | | | **III** | | |
| **Visit** | **1** | **2** | **3** | **4** | **5** | **6**  **SUM IIa** | **7** | **8** | **9**  **SUM IIIa** |
| Driving Behavior Survey - Other  Report |  | X |  | X |  | X |  |  |  |
| BRIEF-A |  | X |  | X |  | X |  |  |  |
| Epworth Sleepiness Scale |  | X | X | X | X | X |  |  | X |
| Beck Anxiety Inventory |  | X |  | X |  | X |  |  | X |
| **Laboratory Measures:** | | | | | | | | | |
| Chemistry e | X |  |  |  |  | X |  |  | X |
| Hematology e | X |  |  |  |  | X |  |  | X |
| Cotinine e | X |  |  |  |  | X |  |  | X |
| TSH e | X |  |  |  |  |  |  |  |  |
| Urinalysis e | X |  |  |  |  | X |  |  | X |
| ECG | X |  |  |  |  | X |  |  | X |
| CYP2D6 f |  |  |  |  |  |  |  |  |  |
| Blood Alcohol Screen e | X |  |  |  |  |  |  |  |  |
| Urine Drug Screen e, g | X |  |  |  |  |  |  |  |  |
| Pregnancy test e, h | X |  |  |  |  |  |  |  |  |

Abbreviations: X = required at this visit; AAQOL-29 = Adult ADHD Quality of Life Scale 29-item; ACDS = Adult ADHD Clinician Diagnostic Scale version 1.2; ASRS = Adult ADHD Symptom Rating Scale; BRIEF-A = Behavior Rating Inventory of Executive Function-Adult Version-Self; CAARS- Inv:SV = Conners’ Adult ADHD Rating Scale-Investigator Rated: Screening Version; CAARS-S:SV = Conners’ Adult ADHD Rating Scale-Self-Report: Screening Version; CGI-ADHD-S = Clinical Global Impressions –ADHD and Severity; MADRS = Montgomery-Asberg Depression Rating Scale; PGI-I = Patient Global Impression – Improvement; PHQ-9 = Patient Health Questionnaire; SASS = Social Adaptation Self-Evaluation Scale ; SCID-RV = Structured Clinical Interview for DSM-IV Axis Disorders-Research Version.

a Study Period II and Study Period III will have summarization packets for the completion or early

discontinuation from each Study Period.

b A waiver with privacy statement and informed consent (ICD) is obtained prior to patient completing the ASRS and inclusion/exclusion questions via website. An additional online ICD is obtained by the investigator site prior to or at Visit 1 if patient is eligible to proceed to the randomized clinical trial. **Consent must be obtained prior to beginning any medication washout required for the sole purpose of entering this study- even if the washout is commenced prior to Visit 1.**

c Presenting/preexisting conditions does not include symptoms of ADHD.

d The Patient-rated outcome measures scales (e-PROS) may be completed via the internet outside of the doctor’s office. Patients may log into the study website 3 days prior to their originally scheduled visit to start the session to complete planned e-PROS. All patient-rated scales must be completed by the end of the scheduled clinic visit.

e If unsuccessful venipunctures occur or if urine samples cannot be obtained, blood and urine samples

may be collected at an additional visit within 10 days without being considered a protocol violation. However, results from clinical lab tests collected at Visit 1 or this additional visit must be received and reviewed prior to dispensing atomoxetine at the end of Visit 2. Repeat or follow-up laboratory tests may be performed at any time at the discretion of the investigator.

f A blood sample may be drawn assayed for CYP2D6 genotype per investigator request in the event of a clinically significant overdose, serious adverse event (SAE), or if a patient is found to have a new onset of neurological signs and symptom. The Lilly Study Physician must be contacted for approval to draw and analyze the sample

g Urine drug screening and blood alcohol screen are required at Visit 1 and may be performed at any other

visit at the investigator’s discretion.

h For females of child-bearing potential, a serum pregnancy test is required at Visit 1 and may be performed at any other visit at the investigator’s discretion.

**Protocol Attachment LYDZ(b).2.**

**Clinical Laboratory Tests**

**Clinical Laboratory Tests**

**Hematologya,b**: **Clinical Chemistrya,b** Hemoglobin **Serum Concentrations of**: Hematocrit Sodium

Erythrocyte count (RBC) Potassium Mean cell volume (MCV) Bicarbonate Leukocytes (WBC) Chloride Neutrophils, segmented Total bilirubin

Neutrophils, juvenile (bands) Alkaline phosphatase

Lymphocytes Gamma-glutamyl transferase (GGT) Monocytes Alanine aminotransaminase (ALT/SGPT) Eosinophils Aspartate aminotransaminase (AST/SGOT) Basophils Blood urea nitrogen (BUN)

Platelets Serum creatinine

Cell morphology Uric acid

Phosphorus

**Urinalysisa,b**: Calcium

Specific gravity Glucose (random) Protein Total protein Glucose Albumin

Blood Cholesterol

Nitrate Creatine kinase (CK) Urine leukocyte esterase

Microscopic examination of sediment **CKMBa,b**

**Serum Cotininea,b**

**Urine Drug Screen a,b,d Serum Pregnancy Test** (females of child- bearing potential only)**a,b,c**

**CYP2D6 enzyme functio**n – whole blood sample**a,f**

**Whole Blood Alcohol a,b,e**

**TSH a,b**

Abbreviations: CYP2D6 = cytochrome P450 2D6; a genetic test for determining CYP2D6 enzyme function (extensive and poor metabolizers).

a Assayed by Lilly-designated Lab.

b Results will be validated by the Central Lab at the time of initial testing. CYP2D6 results will be validated by Lilly during analyses of the data at the end of the study.

c For females of child-bearing potential, a pregnancy test is required at Visit 1 and may be performed at any

other time at the investigator's discretion.

d A urine drug screen is required at Visit 1 and may be performed at any other visit at the investigator's discretion.

e Alcohol testing is required at Visit 1 and may be performed at any other visit at the investigator's

discretion.

f A blood sample may be drawn assayed for cytochrome P450 2D6 (CYP2D6) genotype per investigator request in the event of a clinically significant overdose, serious adverse event (SAE), or if a patient is found to have a new onset of neurological signs and symptom. The Lilly Study Physician must be contacted for approval to draw and analyze the sample.

**Protocol Attachment LYDZ(b).3.**

**Concomitant Medication List**

**Prescription and Over-the-Counter Excluded Drugs (N) and Drugs Allowed (Y) as**

**Concomitant Medications, Study Period I, II, III**

Prescribed and Over-the Counter Drug or Drug Class Episodic Use (as needed) Chronic Use Acetaminophene Ye Ye Allopurinol Y Y Amantadine N N Amiodarone N N Analgesics **f** (non narcotic and except acetaminophen) Yf Yg Anorexics N N Antacids Y N

Anti-inflammatory drugs (nonsteroidal) Y Y Antiasthma agents (except steroids)**a** Y Y Antibiotics (except chloramphenicol) Y Y Anticoagulants N N Anticonvulsants N N Antidepressants N N Antipsychotics N N Antidiarrheal preparations Y N Antiemetics Y N Antihistamines (except diphenhydramine)b Y Y Antihypertensivesc Y Y Benzodiazepines N N Beta-Blockers**h** N Y**h** Calcium Channel blockers Y Y Celecoxib N N Chlorpheniramine N N Cimetidine N N Cough/cold preparations (nonsympathomimetic)**a** Y N Cromolyn sodium Y Y Diphenhydramine Y N Diuretics Y Y Doxorubicin N N

Eszopiclone N N

H2 Blockers (except nizatidine and ranitidine are N N

permitted

Halofantrine N N Haloperidol N N

Health-food supplements that, in the investigator’s N N

opinion, may have central nervous system activity (e.g. St. John’s Wort, melatonin)

Hormones Y Y Indinavir N N Insulin Y Y Laxatives Y Y Lithium N N Narcotics **f** Y N Oral hypoglycemic agents Y Y Oral contraceptives Y Y Table (continues)

**Prescription and Over-the-Counter Excluded Drugs (N) and Drugs Allowed (Y) as**

**Concomitant Medications, Study Period I, II, and III**

| Other psychotropic drugs | N | N |
| --- | --- | --- |
| Psychostimulants | N | N |
| Quinidine | N | N |
| Ranitidine | Y | Y |
| Ritonavir | N | N |
| Ramelteon | N | N |
| Sedative hypnotics  Steroids (oral or inhaled) | N Y**d** | N N |
| Terbinafine | N | N |
| Tryptophan  Zaleplon | N N | N N |
| Zolpidem | N | N |

a Medications may not be used on a daily basis if they are primarily sympathomimetic, e.g., albuterol.

b Antihistamines are not to be used for sedation.

c Clonidine, guanabenz, guanfacine, methyldopa, reserpine, guanethidine, guanadrel, metyrosine, and ketanserin should not be used.

d Short term oral or inhaled steroids are permissible in certain instances including significant allergic

reactions or acute asthma reactions. Please consult the Lilly physician or Lilly designee.

e Use of acetaminophen is limited to 2 grams per day.

f Acute use analgesics and narcotics are allowed if used to treat acute injury or surgical procedure for no longer than 3 days. Narcotics are not allowed during the screening period (before Visit 2).

g Up to 325 mg /day of aspirin is permitted for cardiac prophylaxis.

h Patients may use beta-blockers for treatment of hypertension, but not for treatment of anxiety.

**Prescription and Over-the-Counter (OTC) Medications Prohibited as Concomitant Medications in Protocol B4Z-US-LYDZ(b).**

Use of all concomitant medication (prescription and OTC) must be recorded.

• Concomitant use of monoamine oxidase inhibitors (MAOIs) is never allowed while subjects are taking atomoxetine.

• Atomoxetine is metabolized primarily by cytochrome P450 2D6 (CYP2D6). Many other drugs are metabolized by CYP2D6. Thus, there is the possibility that atomoxetine could alter the metabolism of other

CYP2D6 substrates. Although no such interactions have been observed in

clinical pharmacology studies, when given in combination, plasma concentrations of these drugs and/or concentrations of atomoxetine could be altered. Thus, drugs that are metabolized by CYP2D6 should be used with caution, and the Lilly physician responsible for monitoring the trial should be consulted before such drugs are used in conjunction with atomoxetine. Such drugs include, but are not limited to:

| **SUBSTRATES OF CYP2D6** | |
| --- | --- |
| **CLASS** | **SUBSTRATES** |
| Neuroleptics | perfenazine, thioridazine |
| Antiarrythmic agents | encainide, flecainide, mexiletine, propafenone, sparteine |
| Beta-blockers | Metoprool, propanol, timolol |
| Opioids | codeine, dextromethorphan, hydrocodone, tramadol |
| Others | debrisoquine, perhexiline, phenformin |

Atomoxetine is metabolized primarily by cytochrome P450 2D6 (CYP2D6), and concomitant use of drugs that inhibit this enzyme could result in increased plasma concentrations of atomoxetine. Concomitant administration of atomoxetine and CYP2D6 inhibitors is not permitted in this study. Such drugs include, but are not limited to:

| **INHIBITORS OF CYP2D6** | |
| --- | --- |
| **CLASS** | **INHIBITORS** |
| Antidepressants | clomipramine, fluoxetine, moclobemide, paroxetine |
| Neuroleptics | haloperidol |
| Antiarrythmic agents | amiodarone, quinidine |
| Antiinfectives | halofantrine, ritonavir, terbinafine |
| Others | celecoxib, chlorpheniramine, cimetidine |

More complete lists of CYP2D6 inhibitors and substrates may be found on the internet

[(http://m](http://medicine.iupui.edu/flockhart/))e[dicine.iupui.edu/flockhart/).](http://medicine.iupui.edu/flockhart/)) If in doubt, please contact Lilly.

**Protocol Attachment LYDZ(b).4.**

**Adult Electrocardiogram Exclusion Criteria**

**Eli Lilly and Company**

**Electrocardiogram Exclusion Criteria**

at baseline during study ( if not present at

baseline)

absolute

consult sponsor prior to

discontinue study drug and consult

consult

**ECG Finding** exclusion admission sponsor sponsor

**Rate and Intervals**

Heart Rate ≤40 bpm √ √ Heart Rate ≥130 bpm √ √ QTcBazett’s interval >500 msec √ √

Change from baseline of QTc > 60msec n/a n/a √

**Rhythm**

Junctional Rhythm (JUNCT-R) √ √ Junctional Bradycardia (JUNBRAD) √ √ Junctional Tachycardia (J-TACH) √ √ Undetermined Rhythm (UR) √ √ Atrial Flutter (AFL) √ √ Atrial Fibrilation (AFIB) √ √ Supraventricular Tachycardia (SVT) √ √ Multifocal Atrial Tachycardia (MULT-AT) √ √

Chaotic Atrial Mechanism (CAM) √ √

Unusual P Axis, Possible Ectopic Atrial Rhythms √ √

(EAR)

Bigeminy (BIGEM) √ √

Trigeminy (TRIGEM) √ √

Ventricular Tachycardia or Supraventricular with √ √

Aberration (VTACH)

Wide QRS Tachycardia (WQTACH) √ √ Torsades de Pointes (TOR) √ √ Ventricular Flutter (VFL) √ √ Ventricular Fibrillation (VFIB) √ √ Accelerated Idioventricular Rhythm (AIVR) √ √ With Failure to Capture Consistently (FAILCAPT) √ √

With Failure to Sense Patient’s Own Activity √ √

(FAILSENS)

**Eli Lilly and Company**

**Electrocardiogram Exclusion Criteria (concluded)**

at baseline during study ( if not present

at baseline)

absolute

consult sponsor prior to

discontinue study drug and consult

consult

**ECG Finding** exclusion admission sponsor sponsor

**Conduction**

Sinus Arrest or Block (SAB) √ √

2nd Degree A-V Block (MBZI) √ √

2nd Degree A-V Block (MBZII) √ √ Complete Heart Block (CHB) √ √ AV Dissociation (AVDIS) √ √

**Myocardial Ischemia/Injury/Infarction**

Hyperacute T Wave Abnormality (HYPERT) √ √

ST Depression Consider Ischemia (STDPIN) √ √

Injury Pattern (INJ) √ √

Marked ST Depression, Consider Subendocardial √ √

Injury (SBINJ)

ACUTE MI (ACUMI) √ √

**T Waves**

Tracing Suggests Hypercalcemia (HIGHCA) √ √ Tracing Suggests Hypocalcemia (LOWCA) √ √ Tracing Suggests Hypokalemia (LOWK) √ √ Tracing Suggests Hyperkalemia (HIGHK) √ √

**Additional Findings**

PR Segment Depression, Consider Pericarditis (PRD) √ √

Acute Pericarditis (PCARD) √ √

**Protocol Attachment LYDZ(b).5.**

**Community Sample**

**Introduction and Rationale**

There is a need to better understand the issues of the ADHD complex patient – those with psychiatric co-morbidities. However, to homogenize comparison groups in research clinical trials to facilitate analysis, the complex patients are routinely excluded from clinical trials. As a result, there is a gap between the “real world” clinical patient who may have a complicated clinical presentation and the “simpler patient” that is studied in clinical trials (Rothwell. 2005). The Agency for Healthcare Research and Quality (AHRQ) identified this gap, stating “Studies are also needed to determine whether comorbid factors (for example, anxiety and depressive disorders) influence response to treatment.” (Jadad AR et al. 1999). There have been large epidemiological studies done

to document the extent to which these comorbidities with ADHD exist (Kessler et al.

2006), but little to examine the course of the disorder as a function of these comorbidities.

The community sample (CS) is a superset of subjects who do not qualify or qualify but choose not to participate in the randomized research clinical trial (RCT). Non- interventional health outcomes data via web-based self reporting will be collected via a study website from the CS participants on the same schedule as the RCT participants. For comparative purposes within the community sample, some “trial-like” patients, or patients that are from a screening perspective similar to those who enrolled in the RCT will be recruited. These trial-like patients will be recruited through a central advertising system in cities where a clinical research study site is not available. The participants of

the CS will interface only with the study website and will be independent of the RCT and investigator trial sites. Results from the community sample will be reported separately from the main protocol study report.

**Objectives and the Corresponding Measures**

The primary objective of this CS is to compare the clinical course of ADHD in trial-like and clinically complex patients in a community sample. The “trial-like” group will comprise of respondents who pass the web inclusion/exclusion screen: indicate a willingness to participate in an RCT but do not have a site in their geographic region or declined to participate though a site was available in their region. The “complex” group is defined as subjects who screened positive on the ASRS screener for likelihood of meeting ADHD criteria but who did not otherwise meet the web-based inclusion/exclusion screener for the RCT. The null hypothesis for the CS is: “ADHD patients in the clinically complex group show similar changes over time in ADHD symptom severity as measured by the CAARS-S:SV total ADHD symptom score as compared to patients not in the clinically complex group.”

Secondary objectives include:

• Expansion of prior work regarding treatment selection preferences in typical practice. This would include an assessment of the baseline severity of psychiatric

comorbidities using scores from self report scales (see study schedule of events, Session 1).

• Compare ADHD symptom severity and improvement as measured by the CAARS-S:SV between patient groups with and without presumed comorbidities (for example, anxiety and depression) as measured by self-report of diagnosis, and available scales.

• Compare ADHD symptom severity and improvement as measured by the CAARS-S:SV between patient groups formed by treatment selection (for example, atomoxetine v. alternative).

Secondary objectives are dependent upon sufficient numbers of patients with various comorbidities and treatments enrolling in the CS. There will be no assignment to treatment in this community sample, so it is possible there will be an inadequate sample to address all of these objectives. The sampling frame for the CS is designed around the clinical presentation of the patients, as “trial-like” and “complex”.

**Study Design and Brief Discussion of the Design**

This is a prospective, longitudinal, non-interventional community sample which is comprised of a superset of subjects who do not qualify or qualify but choose not to participate in the randomized research clinical trial (RCT). Subjects eligible for the CS are those who: completed initial web screening for the study, screened positive for the ASRS criteria for ADHD by obtaining a score of 14 or greater on the ASRS v1.1, raw scoring method, but did  **not** qualify for the RCT for any of the following reasons:

• did not meet full inclusion/exclusion for the RCT;

• met full inclusion/exclusion criteria but no site located within their geography; or

• met full inclusion/exclusion criteria and is eligible for RCT but does not wish to participate in the RCT.

Other than the criteria stated above, there are no exclusionary conditions for the CS.1For subjects eligible for the CS and who are interested in participating, the informed consent for the community sample will be obtained via the website prior to collecting any information. Non-interventional health outcomes data via web-based self reporting will be collected via a study website from the CS participants on the same schedule as the RCT participants. A separate schedule of events for the CS is provided in this attachment. Subjects participating in the CS will interface only with a study website and will not be seen by an investigator or clinical trial site. Some additional information on health resource utilization (for example, trips to the emergency room, treatments obtained) will be collected from the CS.

1 While this openness may result in the inclusion of patients with suicidal ideation or terminal illness, such patients exist in clinical practice and excluding them would be counter to the goal of characterizing real-life complexities. Consent forms will clearly indicate that no live or real-time review of forms will occur, nor will health care services be provided. Care will be taken, however, to provide referral information in the event that structured survey responses (there will be no opportunity for free-text responses) suggest increased risk of self-harm. For example, if a patient endorses the PHQ-9 item, indicating “Thoughts that

you would be better of dead or hurting yourself in some way”, the web application will open a window with instructions to contact a health care provider or dial 911 if the person has intent or plan for self harm.

**Power Analysis and Statistical Methods for Analyses**

***Power analysis***

The desired sample size for the CS is based on having sufficient power for detecting clinically relevant differences between the ‘complex’ and ‘non-complex’ populations on changes in the CAARS-S:SV total ADHD symptom score. In order to have 80% power to detect an effect size of 0.2 between these groups, approximately 1,000 subjects must be enrolled. This assumes the imbalance in sample size between the complex and non- complex groups is at most 2:1. Enrollment will be controlled via the web interface to force the enrollment to meet this ratio, if necessary. Also, this assumes that at most 10%

of the enrolled population will not provide follow-up data. While the expected difference

between these groups regarding outcomes such as the CAARS-S:SV is unknown, differences resulting in effect sizes less than 0.2 are considered small.

***Statistical Analysis***

The primary analysis is to compare trial-like and complex patients on ADHD severity (as measured by the CAARS-S:SV) over time. This will be conducted using a mixed model repeated measures (MMRM), with change in CAARS-S:SV total ADHD symptom score as the dependent variable, and Visit (a class variable), baseline CAARS-S:SV total ADHD symptom score, and subject group (trial-like or complex) as independent variables. In addition, this analysis will be repeated utilizing propensity scoring to adjust for multiple other baseline patient characteristics.

Secondary analyses will examine other functional outcome measures and resource utilization using the same contrast groups and statistical methods as for the primary analysis. Logistic regression and the rich clinical and demographic information collected at baseline will be utilized to assess predictors of treatment selection, as a detailed replication and extension of prior work using claims (Van Brunt, 2006). Further secondary analyses, described below, will be conducted as sample sizes for subject subgroups allows. Differences in mean change from baseline in outcome measures between subject groups based on presumed co-morbidities (based on self report of diagnoses and self rating scales) will be assessed using MMRM as for the primary analysis. Similarly, differences between groups based on medication selection will be conducted. Propensity scoring will be utilized to control for baseline differences when comparing these non-randomized groups. Additional exploratory analysis will be conducted as deemed appropriate. Details of the primary and secondary analyses will be documented in a separate statistical analysis plan.

**Schedule of Events**

**Study Schedule for Community Sample**

| **Study Period** | **I** |  | **II** |  |  |  | **III** |  |  |
| --- | --- | --- | --- | --- | --- | --- | --- | --- | --- |
| **Visit** | **1** | **2** | **3** | **4** | **5** | **6**  **SUM II** | **7** | **8** | **9**  **SUM III** |
| **Weeks Until Next Visit:** | **1** | **2** | **3** | **3** | **4** | **2** | **5** | **5** | **N/A** |
| **Interval Between Visits and**  **Target ( )** | **5-28 (7)** | **10-18 (14)** | **17-25 (21)** | **17-25 (21)** | **25-31 (28)** | **10-18 (14)** | **28-38 (35)** | **28-38 (35)** | **-** |
| Privacy Statement* | X |  |  |  |  |  |  |  |  |
| ASRS | X |  |  |  |  |  |  |  |  |
| Inclusion/exclusion criteria  (including PHQ-9) | X |  |  |  |  |  |  |  |  |
| Informed consent* | X |  |  |  |  |  |  |  |  |
| Demographics | X |  |  |  |  |  |  |  |  |
| Social Demographics | X |  |  |  |  |  |  |  |  |
| AAQOL-29 |  | X | X | X | X | X |  |  | X |
| CAARS-S:SV | X | X | X | X | X | X | X | X | X |
| PGI-I |  |  | X | X | X | X |  |  | X |
| Habits Timeline Followback (Alcohol, Nicotine, Caffeine, Marijuana, and Drugs) |  | X | X | X | X | X |  |  | X |
| Fagerstrom Test for Nicotine  Dependence |  | X |  | X |  | X |  |  | X |
| SASS |  | X |  | X |  | X |  |  |  |
| Driving Behavior Survey - Self  Report |  | X |  | X |  | X |  |  |  |
| Driving Behavior Survey - Other  Report |  | X |  | X |  | X |  |  |  |
| BRIEF-A |  | X |  | X |  | X |  |  |  |
| Epworth Sleepiness Scale |  | X | X | X | X | X |  |  | X |
| Beck Anxiety Inventory |  | X |  | X |  | X |  |  | X |
| Resource Utilization (use of medical or psych services or medication) | X | X | X | X | X | X | X | X | X |

* A privacy statement is obtained prior to patient completing the ASRS and an informed consent (ICD) is obtained prior to inclusion/exclusion questions via website. An additional online ICD is obtained prior to or at “Visit 1” if subject is eligible and opts in to proceed to the Community Sample.

Abbreviations: X = required at this visit; AAQOL-29 = Adult ADHD Quality of Life Scale 29-item;

BRIEF-A = Behavior Rating Inventory of Executive Function-Adult Version-Self; CAARS-S:SV = Conners’ Adult ADHD Rating Scale – Self Report: Screening Version; PGI-I = Patient Global Impression – Improvement; PHQ-9 = Patient Health Questionnaire; SASS = Social Adaptation Self- Evaluation Scale.

**References**

Jadad AR, Boyle M, Cunningham C, Kim M, Schachar R. 1999. Treatment of Attention- Deficit/Hyperactivity Disorder. Evid Rep Technol Assess (Summ) No. 11 (Prepared by McMaster University under Contract No. 290-97-0017). AHRQ Publication No 00- E005 Rockville, MD: Agency for Healthcare Research and Quality.

Kessler R, Adler L, Barkley R, Biederman J, Conners CK, Demler O, Faraone SV, Greenhill LL, Howes MJ, Secnik K, Spencer T, Ustun TB, Walters EE, Zaslavsky AM.

2006. The prevalence and correlates of adult ADHD in the United States: results from the National Comorbidity Survey Replication. Am J Psychiatry 163(4):716-723.

Rothwell PM. 2005. External validity of randomized controlled trials: "to whom do the results of this trial apply?". Lancet 365(9453):82-93.

Van Brunt DL, Johnston JA, Ye W, Pohl GM, O’Hara NN. 2006. Factors associated with initiation with atomoxetine versus stimulants in the treatment of adults with ADHD: retrospective analysis of administrative claims data. J Manag Care Pharm 12(3):230-

238.
